# Exploring the impact of physical exercise regimens on health-related quality of life following oesophageal or gastric cancer surgery: a systematic review and meta-analysis of randomized controlled trials

**DOI:** 10.1186/s13102-025-01089-3

**Published:** 2025-03-29

**Authors:** Kenneth Färnqvist, Kalle Mälberg, Asif Johar, Anna Schandl, Monika Fagevik Olsén, Pernilla Lagergren

**Affiliations:** 1 Surgical Care Science, Department of Molecular Medicine and Surgery, Karolinska Institute, Retzius väg 13A, 4th floor, Stockholm, 171 77 Sweden; 2 Department of Anaesthesia and Intensive Care, Södersjukhuset, Stockholm, 118 83 Sweden; 3 Department of Clinical Science and Education, Södersjukhuset, Karolinska Institute, Stockholm, 118 83 Sweden; 4 Department of Surgery & Cancer, Imperial College London, London, UK; 5 Department of Health and Rehabilitation, Institute of Neuroscience and Physiology, Sahlgrenska Academy, University of Gothenburg, Gothenburg, Sweden

**Keywords:** Eesophageal, Gastric, Cancer, Symptoms, Stomach neoplasm, Exercise

## Abstract

**Purpose:**

To assess the effectiveness and adverse events of postoperative physical exercise on health-related quality of life (HRQL) in patients who have undergone surgery for oesophageal or gastric cancer.

**Methods:**

We conducted a systematic review and meta-analysis and reported it according to the preferred reporting items for systematic reviews and meta-analyses. (PRISMA) guidelines. Randomised controlled trials (RCT) that investigated HRQL factors following physical exercise interventions in patients undergoing oesophageal or gastric cancer surgery were included. Studies including participants who had undergone oesophagectomy or gastrectomy for cancer, of either sex and were 18 years or older were included. Participants with other cancers were excluded. Medline, Embase, CINAHL, Cochrane Library, PEDro, and trial registries were searched for studies from inception until February 2025. Results were synthesised using meta-analyses. Two independent reviewers assessed the risk of bias using the Cochrane risk of bias tool 2.0, and the grading of recommendations assessment, development and evaluation (GRADE) was used to evaluate the overall certainty of the evidence. PROSPERO ID CRD42022358493.

**Results:**

Three studies enrolling 284 patients undergoing oesophagectomy were included, of which two were assessed at high risk of bias and one at some concerns. The global quality of life score from the European Organisation for Research and Rreatment of Cancer (EORTC) quality of life questionnaire Cancer QLQ-C30 was used to assess HRQL in all the included studies. The score ranges from 0 to 100, with higher scores indicating a better HRQL. Physical exercise therapy had no discernible impact on HRQL compared to the control group (mean difference 0.77 [95% CI -4.36, 5.90]. However, the quality of evidence was very low, which should be considered when interpreting the results as they can differ substantially from the true effects.

**Conclusion:**

We found a significant lack of information about the effects of post-surgery physical exercise compared to standard care in patients who have undergone oesophagectomy or gastrectomy for cancer. Based on the current very low certainty evidence, the effectiveness on HRQL and the safety of postoperative physical exercise in patients treated with oesophagectomy for cancer is uncertain. We found no studies investigating gastric cancer and exercise.

**Supplementary Information:**

The online version contains supplementary material available at 10.1186/s13102-025-01089-3.

## Introduction

Annually, approximately 1.6 million individuals are diagnosed with oesophagal or gastric malignancies worldwide. Oesophageal cancer is on the rise in the Western world, whereas gastric cancer is declining [1]. For both oesophagal and gastric malignancies, approximately 30–40% of patients meet the criteria for curative-intent therapy, where surgery is the mainstay treatment [2, 3]. Postoperatively, oesophagal and gastric cancer patients experience a variety of adverse effects, including physical symptoms like fatigue, nausea, and pain, as well as psychological issues like depression, anxiety, and social isolation [4–6]. It is worth noting that the severity and duration of these effects may vary depending on when they occur since immediate postoperative effects may differ from those experienced later during the postoperative course [4–6]. These adverse effects can negatively affect a patient’s health-related quality of life (HRQL) by reducing physical and emotional functioning, social and role functioning, and overall well-being [4, 5].

Questionnaires evaluating HRQL assess survivors’ subjective experiences after therapeutic and lifestyle interventions and provide prognostic and predictive information [7] and are, therefore, among the most important measures of cancer survivorship. Exercise interventions have been found to alleviate fatigue [8], improve physical functioning [9], and enhance psychosocial outcomes, such as self-esteem, body image, and social support [10] for various types of cancer. A systematic review and meta-analysis that included study populations with breast cancer (65%), a mixed population (27%), colorectal cancer (9%), and endometrial cancer (3%) found that exercise interventions could positively improve HRQL for up to 60 weeks, particularly physical functioning and role functioning [11].

The literature on exercise following oesophagal or gastric cancer surgery is scarce. A previous systematic review published in 2022, which included both exercise and nutritional interventions, found only one eligible cohort study. This study included 40 patients with advanced gastric cancer who received first-line chemotherapy. The intervention group received standardised nutritional counselling at baseline and every 2 to 4 weeks. The comparison group received counselling at the oncologist’s discretion. No significant differences in overall and progression-free survival were found [12] (no absolute numbers were provided provided). One network meta-analysis examined the impact of exercise on various cancers. The authors concluded that combined (aerobic and resistance) exercise should be recommended as the best option to enhance HRQL during and after cancer treatment [12]. However, only one pre-rehabilitation study on oesophagogastric cancer patients was included, which showed some retention of cardiopulmonary fitness (peak VO2), muscle, and quality of life in prehabilitation participants [13]. Another systematic review [14] investigated physiotherapy regimes after oesophagectomy and gastrectomy for cancer and suggested that exercise interventions may be beneficial in both the preoperative and peri- or postoperative period, however, this review did not follow a pre-registered protocol, a critical flaw according to AMSTAR-2 [15]. The review reported estimated numbers for the standard deviation (SD) for one of the included studies [16], which may have led to incorrect conclusions about consistency and heterogeneity in the outcome of interest (The global quality of life score from the European Organisation for Research and Treatment of Cancer [EORTC] quality of life questionnaire Core-30 [QLQ-C30]). The original publication of the study [16] does not report SD values, so we assume that the review authors calculated the SD from other values reported. Further, the review included studies in which exercise was not studied in isolation and included additional interventions. Lastly, the review did not include a standardised quality/risk of bias assessment (for the assessment of non-randomised trials, no application of the Grading of Recommendations Assessment, Development and Evaluation (GRADE) for the certainty of the evidence), a critical flaw according to AMSTAR-2. With more than one critical flaw, the review “should not be relied on to provide an accurate and comprehensive summary of the available studies” [15]. Due to the uncertainty and methodological limitations in the literature, we wanted to perform a new systematic review considering only randomised controlled trials (RCT) and physical exercise interventions without distorting co-interventions (in exception of the standard of care and breathing exercises). Clinical decisions can then be made regarding potential exercise intervention targets in patients after oesophageal or gastric cancer surgery. We specifically aimed to determine whether postoperative physical exercise implementation compared to a non-exercise intervention affects HRQL in patients who have undergone surgery for oesophageal or gastric cancer.

## Methods

This systematic review was conducted in accordance with the Methodological expectations of Cochrane intervention reviews (MECIR) guidelines [17], and the PRISMA guidelines [18] were used for reporting (checklist presented in Supplementary Table 1). The protocol was registered in the PROSPERO database (CRD42022358493) prior to the study. Before the literature search, we consulted information specialists at the Karolinska Institute Library and decided not only to include full-text articles but also any relevant unpublished data, as this could decrease the risk of publication bias [17]. Therefore, the search strategy was broadened, which differed from that mentioned in the PROSPERO protocol. We also investigated the funding of the individual studies included in the review as an addition to our protocol, as this could affect the outcomes [19]. Adverse events and exercise adherence were added as outcomes to capture the effects of the exercise component more fully. Finally, the Risk Of Bias due to Missing Evidence (ROB-ME) tool [20], released in October 2023, was incorporated for the assessment of bias due to missing evidence, following the publication of our protocol.

### Search strategy

The full search strategies for all databases are available in Supplementary Table 2. A literature search was performed using the following databases: Medline, Embase, CINAHL, and the Cochrane Library. PEDro was used as a complementary database. The PEDro database was searched after completion of the entire search procedure. This was attributable to PEDro’s use of a basic search interface that does not support advanced systematic search techniques, such as Boolean operators, wildcards, or complex nesting. Furthermore, after entering a term, titles and abstracts must be manually screened to identify relevant studies; that is, one cannot export the entire search.

The following trial registries were searched: WHO ICTRP and ClinicalTrials.gov. Eligible registration records were linked to the corresponding publications using their registration numbers, where applicable. In instances of uncertainty regarding the publication status of the trial record, the authors listed in the record were contacted for clarification.

The initial search was conducted on 2023-10-11. The search strategy was developed in Medline (Ovid), in collaboration with librarians at the Karolinska Institute University Library. Medical Subject Headings (MeSH) terms and free text terms were identified for each search concept. The search was then translated, in part, into other databases using Polyglot Search Translator [21]. Databases were searched from inception. Language restriction was made to English. Restricting systematic reviews to English-language publications appears to have little impact on the effect estimates and conclusions of systematic reviews [22].

The strategies were peer-reviewed by another librarian before execution. Deduplication was performed as described by Bramer et al. [23]. One final step was added to compare the Digital Object Identifiers (DOI). A snowball search was conducted to examine the references and citations of all studies included in our review using Citationchaser [24]. The retrieved articles were compared against the original search, and duplicates were eliminated using Rayyan software [25]. The full-text articles remaining after the removal of duplicates were independently reviewed by two reviewers (KF and KM). Any disagreements were resolved through discussion or consultation with the third author (PL). Subsequent to completing the literature search, we contacted via mail 10 individuals within our research network deemed to possess relevant expertise. These individuals were comprised of clinicians, researchers, or a combination thereof. The correspondence provided a concise overview of the review and solicited recommendations for published studies pertinent to our review, as well as any unpublished data. An updated search was conducted on 10 February 2025; however, it did not include searches in the trial registries. We employed the same search strategy but applied a date limitation from 2023 onwards, resulting in some overlapping hits matching those from the initial search.

### Eligibility criteria

#### Types of studies

Only RCT were included in this review to minimise the risk of bias and ensure the robustness of the findings. Eligible studies had to have parallel, single-stage, or crossover designs.

#### Participants

Participants who had undergone oesophagectomy or gastrectomy for cancer, of either sex, and 18 years or older were included. Studies involving participants enrolled with other types of cancer than oesophageal or gastric cancer were excluded.

#### Types of interventions

The physical exercise intervention had to be applied for eight weeks or longer.

The intervention could be implemented either as a standalone intervention or as an addition to the standard of care. The intervention may have been performed as a group exercise or as an individual regime. Physical exercise was defined as “a subset of physical activity that is planned, structured, and repetitive and has as a final or an intermediate objective in the improvement or maintenance of physical fitness” [26]. Consequently, mobility training, strength training, and aerobic/endurance training programs or their combinations were also considered physical exercise. Breathing exercises were accepted as a form of complementary training, as they are frequently recommended in clinical practice.

The interventions could vary in terms of the intensity of the training (low, moderate, high), the length of sessions (minutes), the frequency (sessions/week), and the duration (weeks; a minimum of eight weeks was required). No limitations were imposed on various program attributes, including the setting (hospital, center, or home) or degree of supervision (none, minimal, or observed). Studies that included interventions other than exercise, except for standard care (e.g., massage and cognitive behavioral therapy), were excluded.

The comparator could not include any physical activity/exercise component, except for complementary breathing exercises, as mentioned earlier for the intervention group.

#### Outcome measures

The main outcome of interest was HRQL, which had to be evaluated using validated questionnaires completed by the patients before and after the intervention. Adverse events and adherence to the exercise programs were also examined. Both adverse events and exercise adherence outcomes were analysed and synthesised narratively.

### Study selection

Using Rayyan [25], two independent reviewers (KF and KM) screened all the identified titles and abstracts for inclusion. Full texts of studies retained after title/abstract screening were assessed independently by two authors (KF and KM); disagreements were resolved by discussion or in consultation with a third author (PL). The reasons for the exclusion of studies after full-text review were documented in the supplementary material, page 15.

### Data extraction, synthesis, and statistical analysis

In the table format, two authors (KF and KM) independently extracted the following information from each eligible trial: first author, year of publication, description of each treatment arm, number of participants in each arm, and total number of participants. Further, the following clinical details were extracted: mean age of participants (total and per group); type of cancer (oesophageal/gastric); sex (female/male); type of treatment (e.g., minimally invasive or open surgery); description of the exercise intervention(s) (including the volume and intensity of the entire intervention period, session, adverse events, adherence, and setting [e.g., supervised/unsupervised]); description of the comparative arm; outcome results (at all time points); and the questionnaire used. Continuous outcomes were extracted using raw means and standard deviations when available. Any discrepancies in the extracted data were discussed, and a third author (PL) was involved if a consensus could not be reached. If data relevant to extraction were missing, the authors of the original study were contacted twice over four weeks.

A meta-analysis of trials with similar characteristics would have been optimal, including exercise interventions and comparators, outcome measures, and the study population of interest [17]. However, since clinical and methodological heterogeneity could affect the model findings, especially considering the different exercise types, a random-effects model was planned to be used [27]. Prediction intervals were provided if the random effects model was used [28]. Using prediction intervals, the actual treatment effects expected in a future setting by interpreting clinical heterogeneity were estimated [29]. Where data could not be pooled, a narrative synthesis was used, still using the GRADE approach for the final summary of results [30].

Effect size estimates were calculated when multiple studies examined the same interventions and outcomes. If the results were continuous and the measurement scales were the same, they were expressed as mean differences with 95% confidence intervals (CI). When the outcome measures differed, they were expressed as standardized mean differences (SMD) with 95% CI. Three different types of standard mean difference effect sizes were used: small (0,2), medium (0,5), and large (0,8) [31]. Hedge’s adjusted g [32], which accounts for bias in small samples, was used to adjust the standard mean difference for small samples. Significant results were defined as those with p-values < 0.05.

In cases of non-reported SD, we estimated SD from 95% CI, SE, and p-values. For missing data and SMD cases, we used a t-test, ANOVA, χ² statistics, or p-values to estimate them [33]. A 0–40%, 30–60%, 50–90%, and 75–100% I^2^ value were considered as: might not be important, may represent moderate heterogeneity, may represent substantial heterogeneity, considerable heterogeneity, respectively, as recommended in the Cochrane Handbook for Systematic Reviews of Interventions [17]. Heterogeneity was interpreted as the proportion of variability in effect estimates attributable to between-study dissimilarities rather than within-study sampling uncertainties [34]. If there was reason to believe that there was heterogeneity in the data, possible causes were investigated, including a visual examination of the point estimates and possible underlying factors that may have contributed to the observed heterogeneity [34]. Subgroup analysis was planned for studies on patients treated with chemotherapy and/or radiotherapy before or after surgical treatment, the type of cancer (i.e., oesophageal or gastric), and the type of training (e.g., strength or aerobic). Review Manager 5.4 was used for statistical analyses.

### Study risk of bias assessment and quality assessment

Two independent reviewers (KF and KM) assessed the risk of bias in the studies using the Cochrane risk of bias tool (version 2, ROB2) [35]. Discrepancies were resolved by discussing the entire text until a consensus was reached. The following types of bias were assessed:

- Bias arising from the randomisation process.
- Bias due to deviations from intended interventions (we assessed the effect of assignment to the intervention at baseline, i.e. the ‘intention-to-treat effect’).
- Bias due to missing outcome data.
- Bias in the measurement of the outcome.
- Bias in the selection of the reported results.

The algorithms proposed by ROB2 were subsequently used to categorise each domain into one of the following levels of bias: low risk of bias, some concerns, or high risk of bias.

RevMan 5.4.1 was used to create funnel plots to evaluate the risk of publication bias if more than ten studies per meta-analysis were included, according to the Cochrane Handbook [17]. However, additional studies are probably needed to reach appropriate power [36]. If there were fewer than ten studies, the ROB-ME tool was used [20]. Further, the ROB-Me tool was used to assess the risk of bias that occurs when entire studies or specific results within studies are absent from a meta-analysis due to the P value, magnitude, or direction of the study results (i.e risk of bias due to missing evidence). The assessment was conducted in three stages. 1. Select and define meta-analyses to be assessed. 2. Identify which studies meeting the inclusion criteria for these meta-analyses had missing results. 3. Consider the potential for missing studies in the review. These stages inform the evaluation of the risk of bias related to the missing evidence in a particular meta-analysis. The response options for the signal questions were categorised as ‘yes,’ ‘probably yes,’ ‘probably no,’ ‘no,’ ‘no information,’ or ‘not applicable.’ The results were subsequently interpreted as indicating ‘low risk of bias,’ ‘some concerns,’ or ‘high risk of bias.’ Two independent reviewers (KF and KM) assessed the risk of non-reporting bias, and discussions resolved any discrepancies.

The Grading of Recommendations, Assessment, Development, and Evaluations (GRADE) approach was used to evaluate the overall certainty of evidence [30]. Two authors (KF and KM) independently used the GRADE framework to assess the certainty of evidence. The credibility of Effect Modification Analyses (ICEMAN) in the subgroup analyses was planned to be used [34]. Updated versions have been applied to address inconsistency and imprecision [34, 37]. Funding for individual studies included in the review was also investigated.

## Results

The initial search identified 5059 records, of which 3652 titles and abstracts were screened following deduplication. Following the exclusion of 3,589 studies, including 6 studies we sought to retrieve but did not, 63 full texts were assessed. In total, three studies for quantitative analysis were included [16, 38, 39]. Reasons for exclusion can be found in the Supplementary Material, page 15. The snowball search of the three included articles yielded 117 references, resulting in 111 unique records after the removal of duplicates. Upon comparison with the initial search of 3,652 records, only one record remained for assessment; however, this was subsequently excluded. For the rationale behind this exclusion, refer to supplementary material, page 15.

**Fig. 1:**
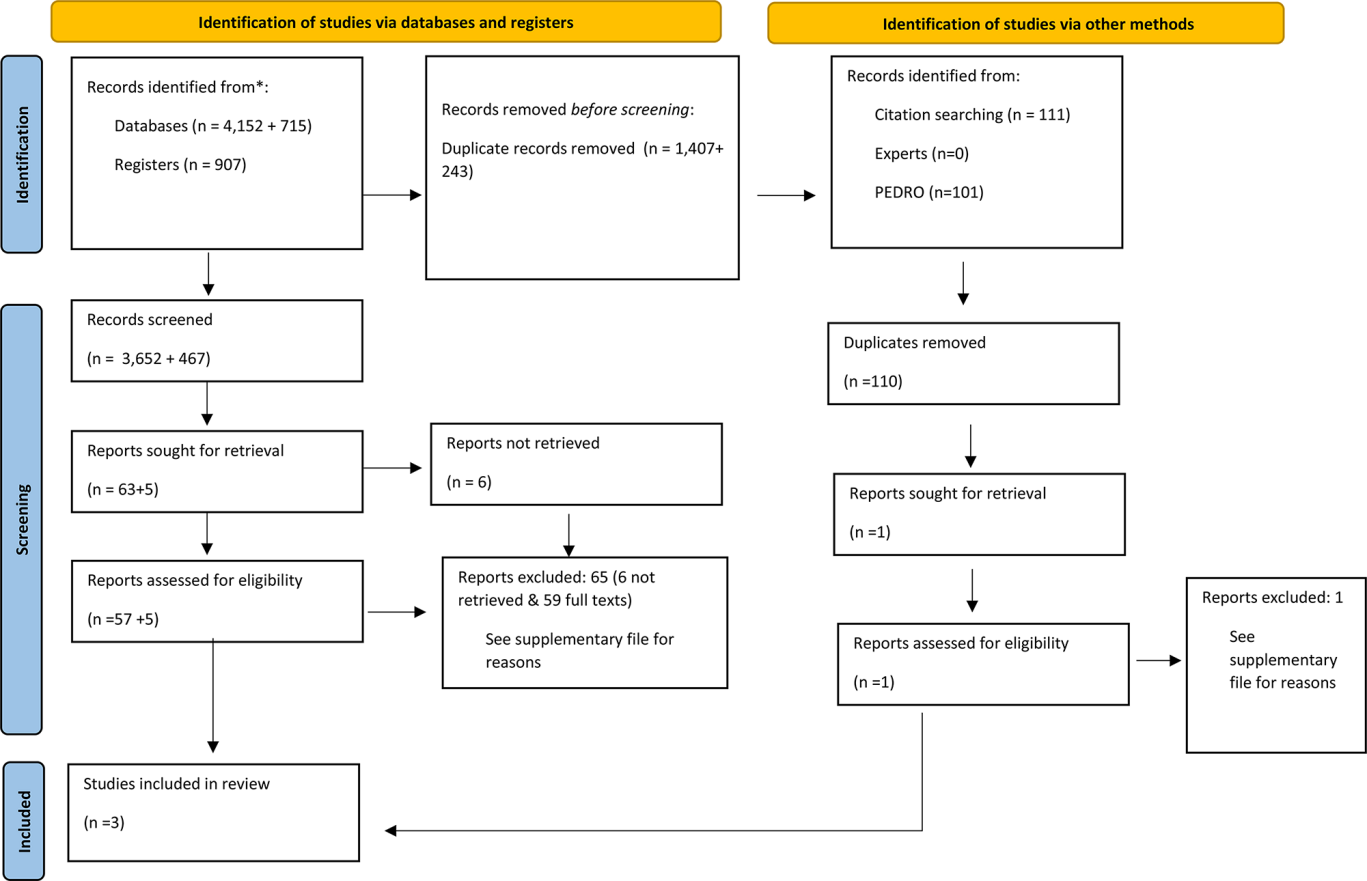
Flow diagram of search strategy. *From*: Page MJ, McKenzie JE, Bossuyt PM, Boutron I, Hoffmann TC, Mulrow CD, et al. The PRISMA 2020 statement: an updated guideline for reporting systematic reviews. BMJ 2021;372:n71. 10.1136/bmj.n71. For more information, visit: http://www.prisma-statement.org/

No relevant studies or data in gray literature, conference abstracts, in contact with experts or reference lists of the included studies were found. However, unpublished data from an RCT [39] published by our research group were included. After this procedure was finalised, the PEDRO database was searched, but no studies of interest were identified. All search strategies can be found in Supplementary Table 2. Figure 1 shows a PRISMA Flow diagram of the search strategy. The updated search identified 715 records, of which 472 titles and abstracts were screened following deduplication. Following the exclusion of 467 studies, five full texts were assessed. All studies were excluded. Reasons for exclusions can be found in the Supplementary material, page 15.

### Characteristics of included studies

Table 1 presents a descriptive summary of the characteristics of the included studies (284 participants).

**Table 1:** Study characteristics described for all enrolled patients at baseline, except for the included number of patients analyzed at follow-up. Pre-operative (preop), postoperative (postop), relative dose intensity (RDI), standard deviation (SD)

Author and year	Number of participants and setting	Age of participants (SD or spread)	Type of cancer	Sex	Type of treatment (e.g. open surgery, minimally invasive)	Intervention and control intervention	Results (only global quality of life score)
Anandavadivelan 2023	Unpublished data Treatment arm: 66 Control arm: 69 Total: 135 The study participants were recruited from within the framework of a nationwide and prospective data collection in Sweden, The Esophageal Surgery in Cancer patients: Adaptation and Recovery study (OSCAR)	Total: 67.5 (59.2–75.8)	Esephoageal Adenocarcinoma and high-grade dysplasia: 110 (84%) Squamous cell Carcinoma: 20 (15%)	Male: 111 (83%) Female: 23 (17%)	Minimally invasive: 50 (38%) Hybrid Thora/ Laparoscopic: 53 (41%) Open esophagectomy: 27 (21%)	The exercise intervention started 1-year post-surgery. Treatment arm: 12-week, home-based exercise training. The research nurse introduced five simple strength training exercises targeting the major muscle groups with repetitions of 10 and 2 sets to do twice a week. The resistance training was progressive in nature by means of increasing resistance in the band during the 12 weeks if acceptable with the patients and depended on their adaptability to the intervention. The research nurse suggested 150 min weekly activity of minimum intensity (e.g., walking, swimming, cycling) to the patients. Control arm: Standard of care + informed about the WHO recommendations for physical activities among older people (150 min weekly and strength training two times weekly) and their health benefits	Unpublished data QLQ-C30 Global quality of life Intervention group baseline 71.21 (23.58) 12 weeks 70.13 (21.75) Control group baseline 69.44 (18.45) 12 weeks 67.52 (20.47) Adverse events not reported Exercise adherence was followed up weekly by telephone. Results not reported
Fagevik 2017	Treatment arm: 20 Control arm: 23 Total: 43 Patients scheduled for Ivor-Lewis esophageal resections at Sahlgrenska University Hospital	Total: 62.5	Esophageal cancer	Male: 35 (81%) Female: 8 (19%)	Ivor-Lewis resection of the esophagus (100%)	The exercise intervention on the first day after surgery Treatment arm: All patients met a specialized physical therapist before discharge for a discussion about their normal physical activity level and how to regain this function. The patients in the intervention group also received a rehabilitation program that consisted of three leaflets of exercises to restore lung function, range of motion in the thoracic spine and shoulder, and strength exercises for the back extensors, shoulders, and legs. The program was gradually increased in intensity through the rehabilitation period. All exercises were described in detail and the patients carried out the exercises in the first program under supervision. The patients were encouraged, unsupervised at home from the day of discharge, to perform the range of motion daily until they felt no restrictions or pain at the end range. Control arm: The control group was given the information that it is not known whether any training may be beneficial and was told to avoid specific interventions during the first three months postoperatively. Those discharged to convalescence care received no specific additional instructions or exercises to improve rib cage or lung function.	QLQ-C30 Global quality of life Intervention group baseline 72.4 (19.5) 12 weeks 61.6 (20.3) Control group baseline 65.9 (26.3) 12 weeks 70.1 (22.5) A training diary was kept to record exercise adherence. Results not reported No side effects of the intervention were recorded
Van vulpen 2021	Treatment arm: 54 Control arm: 56 Total: *n* = 110 The study was conducted in nine Dutch hospitals	Total: 63.7 (8.1)	Esophageal Adenocarcinoma: 92 (77%) Squamous cell carcinoma 20 (17%) Adenosquamous 1 (> 1%) Other 7 (6%)	Male: 104 (87%) Female: 16 (13%)	Open esophagectomy: 11 (9%) Thoraco-laparoscopic: 55 (46%) Transhiatal-laparoscopic: 7 (6%) Robot-assisted: 47 (39%)	The exercise intervention started 4–52 weeks after hospital discharge following Surgery Treatment arm: the program included two combined aerobic and resistance training exercise sessions per week, supervised by an outpatient (oncology) physiotherapist. The 60-minute exercise sessions included a warm-up (5 min), aerobic and resistance training (50 min), and a cooling down (5 min) period. In addition to the supervised exercise program, participants were asked to be physically active for at least 30 min/per day on all remaining days of the week. Control arm: Standard care	QLQ-C30 Global quality of life Intervention group baseline 67.48 (17.19) 12 weeks 77.16 (17.37) Control group baseline 71.05 (16.26) 12 weeks 75.00 (17.91) The between-group difference at 24 weeks with the control group as a reference − 2.86 (CI − 8.37, 2.65). Adherence to the protocol was evaluated by recording session attendance and adherence to the planned dose/session. Deviations from the scheduled exercise dose were recorded by the physiotherapist 96% participation rate in exercise sessions. RDI for moderate- to high-intensity endurance exercises, high-intensity endurance exercises, interval training, and resistance exercises was 94 (i.q.r. 87–100), 90 (i.q.r. 70–100), 100 (i.q.r. 74–100) and 90 (i.q.r. 81–97) percent, respectively. The RDI of the exercise advice, that is, being active 7 days of the week or 5 days/week (Dutch Physical Activity Guidelines) for 12 weeks was 25 (i.q.r. 0–58) and 75 (i.q.r. 17–92) percent, respectively. No exercise-related serious adverse events were observed

### Participants

The analyses involved three trials with 284 participants with an average age of 65 years. 84% of the participants were male. All participants had undergone oesophagectomy, with the majority (81%) having oesophagal adenocarcinoma. However, Fagevik Olsén et al. [38] did not specify the type of cancer.

### Interventions

The analysis encompassed three studies comprising three treatment and three control groups. Of these, one included a strength-based intervention [39], and the other two [16, 38] included mixed types of physical exercise (e.g., mobility, strength, and aerobic). Physical exercise interventions were individually supervised in one study [16], and the other two included home-based training [38, 39]. The volume (sets/reps) and intensity were not described in detail in two studies [39, 38], but the frequency was described in all studies [16, 39, 38]. The comparison intervention in all studies comprised the standard of care, except for advice to stay active [16, 39]. As per the eligibility criteria, no study included additional treatment with exercise interventions.

### Outcomes

The global quality of life (GQOL) score within the QLQ-C30 was used to assess HRQL in all included studies. After emailing the last author, we obtained raw data on follow-up scores with SD, as this was not reported in the original publication [16]. The GQOL scores from the study by Anandavadivelan et al. [39] were obtained from an experienced biostatistician (AJ) within our research group, as the GQOL scores constitute a pre-specified secondary analysis from the published RCT but remain unpublished at present. Adverse effects or side effects were measured in two studies [16, 38]; however, no pre-registered definitions were made in any of the studies.

Exercise adherence was measured using an exercise diary [38], by a physiotherapist supervising the exercise session [16], and by telephone [39].

### Risk of bias in included studies and due to missing evidence, and GRADE assessment

**Fig. 2:**
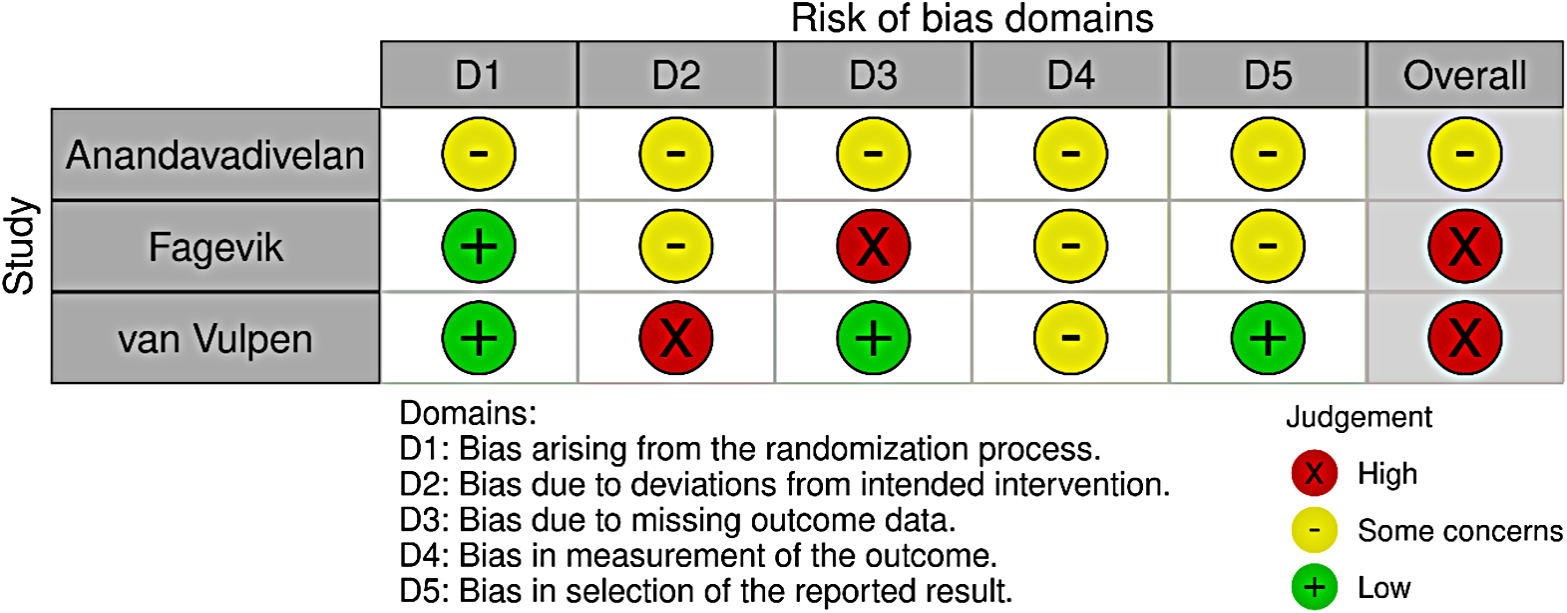
Risk of bias assessment of the individual included studies

**Fig. 3:**
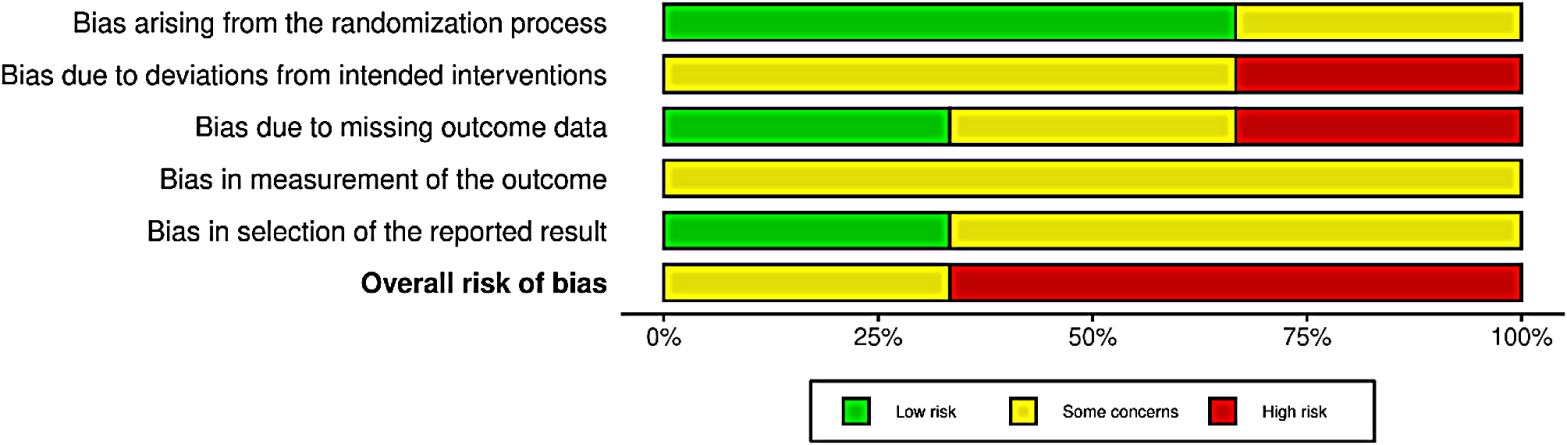
Summary of risk of bias judgments of the included studies for HRQL. Figures 2 and 3 were created using the Robvis web app. 1. McGuinness LA, Higgins JPT. Risk-of-bias VISualization (robvis): An R package and Shiny web app for visualising risk-of-bias assessments. Research Synthesis Methods. 2020

None of the included studies had a low risk of bias (Fig. 2). Two were assessed as high-risk [16, 38], and one had some concerns [39]. The domains with the highest risk of bias were the risk of deviation from the intended intervention and measurement of the outcome. Conversely, the domain of bias arising from the randomisation process showed the least risk, with two studies having a low risk and one with some concerns (Fig. 3). All included studies reported funding sources with no apparent concerns.

The studies that were included in the analysis did not have any missing results (although we requested the SD reported in the van Vulpen et al. study, as they were not reported in their publication). We extracted only one specific outcome measure from a particular questionnaire and there was no evidence of non-reporting bias. For the across-study assessment, the circumstances did not indicate the potential for missing studies, and the pattern of results suggests that there are unlikely to be missing results that are systematically different from those observed. One study [40] was excluded because it was not reported in English. This study found a significant difference in HRQL between the exercise and control groups for patients who had undergone surgery for gastric cancer but were still under oral chemotherapy. The study included 23 patients, and the difference between the groups was 0.79 points on a 14-70-point scale (unvalidated scale, used in an unpublished master’s thesis). When including the study in the meta-analysis, the standardised mean difference (the study used a different type of HRQL questionnaire) was still not significant using a random-effects model (figure shown in the supplementary file). The certainty of the evidence for HRQL is presented in Table 2. The certainty of evidence was downgraded due to the risk of bias and indirectness, resulting in a very low certainty of evidence.

**Table 2:** Summary of evidence table

**People**: Patients who have undergone surgery for esophageal cancer (no studies on gastric cancer patients were included)				
**Settings**: Sweden and the Netherlands				
**Intervention**: Exercise				
**Comparison**: Control				
**Outcomes**	**Pooled mean difference**	**Number of studies**	**Certainty** **of the evidence** **(GRADE)**	**Comments**
HRQL	0.77 [-4.36, 5.90]	3	⊕⊖⊖⊖ Very low^1^	It is uncertain whether exercise leads to higher HRQL because the certainty of the evidence is very low

**Very low** = This research does not provide a reliable indication of the likely effect. The likelihood that the effect will be substantially different^‡^ is very high

^‡^ Substantially different = a large enough difference that it might affect a decision

^1^We downgraded the certainty of evidence by two due to risk of bias (two out of three studies rated as high risk of bias) and due to indirectness (limited number of studies for each exercise type)

### Effects of physical exercise

**Fig. 4:**
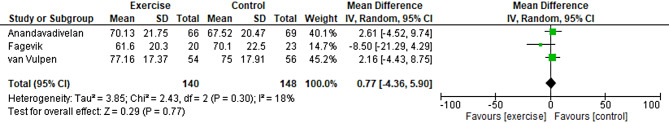
Random effect model. Effect of exercise on health-related quality of life. CI, confidence interval; df, degrees of freedom; IV, inverse variance; SD, standard deviation

Table 2 summarises the findings of the comparison between physical exercise and standard treatments. The complete meta-analyses data are shown in Fig. 4. Regarding HRQL, no evidence of a difference between physical exercise and standard treatment was observed. It is uncertain whether physical exercise leads to any difference in GQOL because the certainty of the evidence was very low.

A total of 284 participants were included in the analysis to compare the effects of physical exercise and standard treatment on HRQL for patients following oesophagectomy. The results revealed that physical exercise treatment had a pooled mean difference of 0.77 [95% CI -4.36, 5.90] compared with no treatment (Fig. 4), with a prediction interval of [− 8.36, 9.90]. The results showed that physical exercise did not lead to any significant improvement in GQOL outcomes. The evidence supporting the comparison of physical exercise treatment with the standard of care was considered very low due to the substantial risk of bias (downgraded by two levels) and indirectness (downgraded by one level). Study authors might consider using a fixed effects model, which is deemed more appropriate given the low values of I^2^ and the inclusion of few studies [41]. This approach would provide a better estimate of the common intervention effect across studies [42]. However, we have three studies presenting different exercise interventions at different time points post-surgery and therefore deemed a random-effects model more appropriate [41]. Although subgroup analyses were planned, this was not possible because of the small number of studies included. However, we found relatively low heterogeneity, and for all possible combinations of only two studies in a meta-analysis, no significant results were obtained (data not shown).

Table 1 shows the results of adverse events and exercise adherence. No adverse events were reported. The study by van Vulpen [16] showed excellent adherence, while the other two studies [38, 39] did not have adherence data.

## Discussion

This systematic review and meta-analysis summarize recent literature on postoperative physical exercise and its effect on HRQL in patients who have undergone oesophagectomy or gastrectomy for cancer. This review included three studies and found that exercise therapy had no discernible impact on HRQL compared to standard treatment for patients following oesophagectomy. However, the quality of evidence supporting this finding was very low, necessitating extreme prudence when interpreting the results, as they likely differ substantially from the true effects. A paucity of comprehensive data was uncovered, which hinders the ability to perform a rigorous analysis of structured physical exercise interventions for oesophageal or gastric cancer patients undergoing surgery. Furthermore, no studies investigating patients with gastric cancer were found.

This study had some limitations. Regarding the limitations of the review process, we made some changes during the post-registration phase; the protocol was changed, including the decision to include full-text articles and relevant unpublished data. These changes were made prior to the literature search and data collection. Furthermore, we limited publications to English, which resulted in the exclusion of one study that investigated postoperative exercise in patients with gastric cancer who were undergoing oral chemotherapy [40]. This represents a significant limitation, as we only included three studies, all of which investigated patients with oesophagal cancer.

When it comes to limitations regarding the evidence base, the primary limitations were related to the small number of included studies, their high risk of bias, and their absence of statistical power, which led to the conclusion that the estimate of effect was very uncertain. It is important to emphasize the complete absence of publications examining exercise following gastric cancer; consequently, the findings should not be extrapolated to that specific population. As reflected in the risk of bias assessment, it is important to note that blinding exercise interventions are difficult, both when it comes to participants, providers, and assessors. This means that patients may be susceptible to performance and ascertainment biases, thereby overestimating the results [43]. For this review, outcomes were reported by the participants, and the outcome assessor was considered the study participant. For participant-reported outcomes, the assessment of outcomes may be influenced by knowledge of the intervention received, potentially introducing bias in reporting [35]. However, the conclusions do not change because the certainty of the evidence is more important than the effect estimates. To further elaborate on the effects of physical exercise, the reporting of physical exercise adherence was deficient. This must be considered a significant flaw, as the reported information did not reveal anything about intensity or volume [44], except for one study [16]. Other measurements also encouraged to be reported in trials including physical exercise are motivation to perform the physical exercises or knowledge about why they perform the physical exercises, which guidelines and supporting documents recommend [45, 46].

Adverse effects or side effects were measured in two studies [16, 38]. However, no measurement of adverse effects was mentioned other than exercise/intervention-related and was not described in the Methods section, but only as a single sentence in the Results section. One of the study protocols mentioned the measurement of adverse effects, with only the presence or absence of adverse events and no definition of either [47]. Given the absence of data, it is impossible to make any assertions regarding the presence or severity of any adverse effects, including even mild effects such as muscle soreness, which may have gone unreported within the missing classifications. Based on the results of a systematic review and meta-analysis by Thomsen et al. [48], it appears that there may be a heightened likelihood of harm associated with exercise in cancer patients undergoing systemic treatment. However, the authors maintain that the evidence is inconclusive and that there is insufficient data available to comprehensively evaluate the risks and benefits of structured exercise in this population.

It is not feasible to apply a single, universally applicable, and minimally clinically important difference across all QLQ-C30 scales and various disease conditions. Consequently, caution should be exercised when employing generalised guidelines in such contexts [49]. However, interpreting the results in our review with a change of less than one point on a 100-point scale, disregarding the weak evidence, it is evident that this cannot be considered a meaningful change. According to a systematic review and network meta-analysis by Martínez-Vizcaíno et al. [12], all types of exercise can positively affect HRQL in various types of cancer, both during and after treatment. However, the data indicated that only combined exercise significantly affected the patients during treatment. Although the evidence behind the recommendation is weak, the authors agree with the international consensus and guidelines that exercise should be recommended to improve HRQL in cancer patients [50].

Physical exercise has been proposed as a potential method to enhance HRQL, with several systematic reviews of various cancer types demonstrating positive outcomes [12, 51]. However, limited research has investigated the mediators of the effects of exercise on HRQL in cancer survivors [52–56]. These studies indicate that the relationship between improved cardiorespiratory fitness and enhanced HRQL is mediated by fatigue. Additionally, psychological constructs have been observed to play a mechanistic role, with increased self-efficacy and positive affect resulting from physical activity interventions significantly associated with improved HRQL [57]. On the other hand, contextual factors, including physical, psychological, and social elements that characterise the therapeutic encounter with the patient, but also their living environment (e.g. urban or rural), can induce placebo or nocebo effects. Consequently, these effects may obscure the veridical effect of the treatment [58, 59]. Therefore, elucidation of the mechanisms that connect physical activity to cancer is of utmost importance to enhance our understanding of the disease and to devise efficacious strategies to combat it. Gaining insight into these mechanisms can provide evidence to support the implementation of physical activity programs at various levels and provide a basis for the development of prevention and treatment methods. Furthermore, this knowledge can serve to confirm research results and contribute to the advancement of cancer biology. If such evidence shows that increased physical activity or a certain type of exercise can prevent certain cancers or improve prognosis, it could be a valuable public health intervention with low cost and risk [60].

Numerous RCT have focused on investigating the effectiveness of physical exercise as an intervention to optimise patients’ physical status prior to oesophageal and gastric cancer treatment. However, only one systematic review published in 2021 by Tukanova [14] has examined the impact of exercise post-surgery in patients with oesophageal or gastric cancer, focusing on HRQL. Still, this study suffered from a few shortcomings, such as not following a pre-registered protocol, included studies in which exercise was not studied as a standalone treatment, and not including a standardised quality/risk of bias assessment. The present systematic review provides the most comprehensive evaluation of evidence based on the effectiveness of physical exercise treatment, without the use of any adjuncts, in patients who have undergone surgery for oesophageal or gastric cancer.

## Conclusions

This systematic review and meta-analysis highlight a significant gap in the literature regarding the effects of post-surgery physical exercise in comparison to standard care for patients who have undergone oesophagectomy or gastrectomy. Based on the current very low certainty evidence, the effectiveness on HRQL and the safety of postoperative physical exercise in patients treated with oesophagectomy for cancer is uncertain. We found no studies investigating gastric cancer and exercise. Further research is required to determine the potential benefits or harms of exercise interventions in patients who have undergone surgery for oesophageal or gastric cancer. It is imperative to conduct well-designed RCT to evaluate the potential benefits and risks of exercise in this patient population.

## Electronic supplementary material

Below is the link to the electronic supplementary material.

Supplementary Material 1

## Data Availability

See the accompanying supplementary file for further details.
