# Supplementary material for: Exploring the impact of physical exercise regimens on health-related quality of life following oesophageal or gastric cancer surgery: a systematic review and meta-analysis of randomized controlled trials
Source: BMC Sports Sci Med Rehabil. 2025 Mar 29;17:64. doi: 10.1186/s13102-025-01089-3 (PMC11955135; doi:10.1186/s13102-025-01089-3)
Supplement: Supplementary file 1 — Supplementary Material 1 [file 13102_2025_1089_MOESM1_ESM.docx]

**Supplementary materials**

**Exploring the Impact of Physical Exercise Regimens on health-related quality of life following esophageal or gastric cancer surgery: a systematic review and meta-analysis of randomized controlled trials.**

**Supplementary Table 1.** PRISMA checklist.

| **Section and Topic** | **Item #** | **Checklist item** | **Location where item is reported** |
| --- | --- | --- | --- |
| **TITLE** | | |  |
| Title | 1 | Identify the report as a systematic review. | 1 |
| **ABSTRACT** | | |  |
| Abstract | 2 | See the PRISMA 2020 for Abstracts checklist. | 2 |
| **INTRODUCTION** | | |  |
| Rationale | 3 | Describe the rationale for the review in the context of existing knowledge. | 3 |
| Objectives | 4 | Provide an explicit statement of the objective(s) or question(s) the review addresses. | 4-5 |
| **METHODS** | | |  |
| Eligibility criteria | 5 | Specify the inclusion and exclusion criteria for the review and how studies were grouped for the syntheses. | 6-7 |
| Information sources | 6 | Specify all databases, registers, websites, organizations, reference lists, and other sources searched or consulted to identify studies. Specify the date when each source was last searched or consulted. | 5-6 (sup. Table 2, fig 1) |
| Search strategy | 7 | Present the full search strategies for all databases, registers, and websites, including any filters and limits used. | Sup. Table 2 |
| Selection process | 8 | Specify the methods used to decide whether a study met the inclusion criteria of the review, including how many reviewers screened each record and each report retrieved, whether they worked independently, and if applicable, details of automation tools used in the process. | 8 |
| Data collection process | 9 | Specify the methods used to collect data from reports, including how many reviewers collected data from each report, whether they worked independently, any processes for obtaining or confirming data from study investigators, and if applicable, details of automation tools used in the process. | 8-9 |
| Data items | 10a | List and define all outcomes for which data were sought. Specify whether all results that were compatible with each outcome domain in each study were sought (e.g. for all measures, time points, analyses), and if not, the methods used to decide which results to collect. | 8-9 |
|  | 10b | List and define all other variables for which data were sought (e.g. participant and intervention characteristics, funding sources). Describe any assumptions made about any missing or unclear information. | 8-9, table 1 |
| Study risk of bias assessment | 11 | Specify the methods used to assess the risk of bias in the included studies, including details of the tool(s) used, how many reviewers assessed each study and whether they worked independently, and if applicable, details of automation tools used in the process. | 10 |
| Effect measures | 12 | Specify for each outcome the effect measure(s) (e.g. risk ratio, mean difference) used in the synthesis or presentation of results. | 8-9 |
| Synthesis methods | 13a | Describe the processes used to decide which studies were eligible for each synthesis (e.g. tabulating the study intervention characteristics and comparing against the planned groups for each synthesis (item #5)). | 8-9 |
|  | 13b | Describe any methods required to prepare the data for presentation or synthesis, such as handling of missing summary statistics, or data conversions. | 8-10 |
|  | 13c | Describe any methods used to tabulate or visually display results of individual studies and syntheses. | 9-10 |
|  | 13d | Describe any methods used to synthesize results and provide a rationale for the choice(s). If meta-analysis was performed, describe the model(s), method(s) to identify the presence and extent of statistical heterogeneity, and software package(s) used. | 9-10 |
|  | 13e | Describe any methods used to explore possible causes of heterogeneity among study results (e.g. subgroup analysis, meta-regression). | 8-10 |
|  | 13f | Describe any sensitivity analyses conducted to assess the robustness of the synthesized results. | 8-10 |
| Reporting bias assessment | 14 | Describe any methods used to assess the risk of bias due to missing results in a synthesis (arising from reporting biases). | 10 |
| Certainty assessment | 15 | Describe any methods used to assess certainty (or confidence) in the body of evidence for an outcome. | 10 |
| **RESULTS** | | |  |
| Study selection | 16a | Describe the results of the search and selection process, from the number of records identified in the search to the number of studies included in the review, ideally using a flow diagram. | 10-11 |
|  | 16b | Cite studies that might appear to meet the inclusion criteria, but which were excluded, and explain why they were excluded. | 10, sup material |
| Study characteristics | 17 | Cite each included study and present its characteristics. | Table 1 |
| Risk of bias in studies | 18 | Present assessments of risk of bias for each included study. | 12-13, fig 2-3 |
| Results of individual studies | 19 | For all outcomes, present, for each study: (a) summary statistics for each group (where appropriate) and (b) an effect estimate and it's precision (e.g. confidence/credible interval), ideally using structured tables or plots. | 10-12, fig, table 1- 2 |
| Results of syntheses | 20a | For each synthesis, briefly summarise the characteristics and risk of bias among contributing studies. | 12-13, table 2 |
|  | 20b | Present results of all statistical syntheses conducted. If meta-analysis was done, present for each the summary estimate and its precision (e.g. confidence/credible interval) and measures of statistical heterogeneity. If comparing groups, describe the direction of the effect. | 13-14, fig 4, table 2 |
|  | 20c | Present results of all investigations of possible causes of heterogeneity among study results. | 13-14, fig 4, table 2 |
|  | 20d | Present results of all sensitivity analyses conducted to assess the robustness of the synthesized results. | 13-14 |
| Reporting biases | 21 | Present assessments of risk of bias due to missing results (arising from reporting biases) for each synthesis assessed. | 12-13 |
| Certainty of evidence | 22 | Present assessments of certainty (or confidence) in the body of evidence for each outcome assessed. | 13-14, Table 2 |
| **DISCUSSION** | | |  |
| Discussion | 23a | Provide a general interpretation of the results in the context of other evidence. | 14 |
|  | 23b | Discuss any limitations of the evidence included in the review. | 14-15 |
|  | 23c | Discuss any limitations of the review processes used. | 14-15 |
|  | 23d | Discuss the implications of the results for practice, policy, and future research. | 16-17 |
| **OTHER INFORMATION** | | |  |
| Registration and protocol | 24a | Provide registration information for the review, including register name and registration number, or state that the review was not registered. | 5 |
|  | 24b | Indicate where the review protocol can be accessed, or state that a protocol was not prepared. | 5 |
|  | 24c | Describe and explain any amendments to information provided at registration or in the protocol. | 5 |
| Support | 25 | Describe sources of financial or non-financial support for the review, and the role of the funders or sponsors in the review. | 18 |
| Competing interests | 26 | Declare any competing interests of review authors. | 18 |
| Availability of data, code, and other materials | 27 | Report which of the following are publicly available and where they can be found: template data collection forms; data extracted from included studies; data used for all analyses; analytic code; any other materials used in the review. | 18 |

**Supplementary table 2.** Full search strategy

1. Medline

| Interface: Ovid MEDLINE(R) ALL  Date of Search: 11 October 2023  Number of hits: 987  Comment: In Ovid, two or more words are automatically searched as phrases; i.e. no quotation marks are needed | Field labels   - exp/ = exploded MeSH term - / = non exploded MeSH term - .ti,ab,kf. = title, abstract, and author keywords - adjx = within x words, regardless of order - * = truncation of word for alternate endings |
| --- | --- |
| Database(s): **Ovid MEDLINE(R) ALL**1946 to October 10, 2023 Search Strategy:   \| **#** \| **Searches** \| **Results** \| \| --- \| --- \| --- \| \| 1 \| exp Esophageal Neoplasms/ \| 59560 \| \| 2 \| Gastrointestinal Neoplasms/ \| 20279 \| \| 3 \| Stomach Neoplasms/ \| 111322 \| \| 4 \| ((abdomen or abdominal or esophag* or gastric or gastroesophage* or gastrointestin* or oesophag* or stomach*) and (adenocarcinoma* or carinoma* or cancer* or malignan* or neoplas* or tumor* or tumour)).ti,kf. \| 147864 \| \| 5 \| ((abdomen or abdominal or esophag* or gastric or gastroesophag* or gastrointestin* or oesophag* or stomach*) adj3 (adenocarcinoma* or carinoma* or cancer* or malignan* or neoplas* or tumor* or tumour*)).ab. \| 160472 \| \| 6 \| or/1-5 \| 264860 \| \| 7 \| Digestive System Surgical Procedures/ \| 21188 \| \| 8 \| Postoperative Care/ \| 60821 \| \| 9 \| Surgical Procedures, Operative/ \| 56875 \| \| 10 \| surgery.fs. \| 2270976 \| \| 11 \| (operat* or postoperat* or postop or post-operat* or post-op or resect* or surgery or surgical* or surgeries).ti,ab,kf. \| 3487354 \| \| 12 \| or/7-11 \| 4410309 \| \| 13 \| 6 and 12 \| 101567 \| \| 14 \| Esophagectomy/ \| 12639 \| \| 15 \| Gastrectomy/ \| 41017 \| \| 16 \| Gastroenterostomy/ \| 3660 \| \| 17 \| (esophagectom* or gastrectom* or gastroenterostom* or oesophagectom*).ti,ab,kf. \| 54416 \| \| 18 \| or/14-17 \| 72975 \| \| 19 \| 13 or 18 \| 138509 \| \| 20 \| Exercise/ \| 143462 \| \| 21 \| exp Exercise Therapy/ \| 63935 \| \| 22 \| exp Physical Conditioning, Human/ \| 18145 \| \| 23 \| exp Physical Fitness/ \| 36534 \| \| 24 \| Physical Therapy Modalities/ \| 41031 \| \| 25 \| (exercis* or kinesiotherap* or physical function* or physical therap* or physiotherap* or physio-therap* or training program*).ti,ab,kf. \| 495226 \| \| 26 \| (physical* adj3 (activ* or conditioning* or fit or inactiv* or intervention* or program*)).ab. \| 171100 \| \| 27 \| (physical* and (activ* or conditioning* or fit or inactiv* or intervention* or program*)).ti,kf. \| 80693 \| \| 28 \| (fitness adj3 (cardiorespirat* or physical* or training*)).ab. \| 18849 \| \| 29 \| (fitness and (cardiorespirat* or physical* or training*)).ti,kf. \| 13323 \| \| 30 \| (training adj3 (endurance* or intervent* or physical*)).ab. \| 24389 \| \| 31 \| (training and (endurance* or intervent* or physical*)).ti,kf. \| 17793 \| \| 32 \| or/20-31 \| 710310 \| \| 33 \| 19 and 32 \| 1095 \| \| 34 \| limit 33 to english language \| 987 \| | |

2. Embase

| Interface: embase.com  Date of Search: 11 October 2023  Number of hits: 2,382  Comment: Emtree is the controlled vocabulary in Embase | Field labels   - /exp = exploded Emtree term - /de = non exploded Emtree term - ti,ab,kw = title, abstract and author keywords - NEAR/x = within x words, regardless of order - * = truncation of word for alternate endings |
| --- | --- |
| \| **No.** \| **Query** \| **Results** \| \| --- \| --- \| --- \| \| #1 \| 'esophagus cancer'/de OR 'esophagus carcinoma'/exp \| 90,077 \| \| #2 \| 'gastrointestinal tumor'/de OR 'gastrointestinal stromal tumor'/de OR 'gastrointestinal cancer'/de OR 'gastrointestinal carcinoma'/de OR 'gastrointestinal adenocarcinoma'/de \| 39,562 \| \| #3 \| 'stomach cancer'/de OR 'stomach carcinoma'/exp \| 149,357 \| \| #4 \| (abdomen:ti,kw OR abdominal:ti,kw OR esophag*:ti,kw OR gastric:ti,kw OR gastroesophage*:ti,kw OR gastrointestin*:ti,kw OR oesophag*:ti,kw OR stomach*:ti,kw) AND (adenocarcinoma*:ti,kw OR carinoma*:ti,kw OR cancer*:ti,kw OR malignan*:ti,kw OR neoplas*:ti,kw OR tumor*:ti,kw OR tumour:ti,kw) \| 198,832 \| \| #5 \| ((abdomen OR abdominal OR esophag* OR gastric OR gastroesophage* OR gastrointestin* OR oesophag* OR stomach*) NEAR/3 (adenocarcinoma* OR carinoma* OR cancer* OR malignan* OR neoplas* OR tumor* OR tumour)):ab \| 229,026 \| \| #6 \| #1 OR #2 OR #3 OR #4 OR #5 \| 368,470 \| \| #7 \| 'abdominal surgery'/de OR 'esophagus surgery'/de OR 'stomach surgery'/de \| 49,196 \| \| #8 \| 'postoperative care'/de \| 116,442 \| \| #9 \| 'surgery'/de \| 807,973 \| \| #10 \| surgery:lnk \| 2,557,411 \| \| #11 \| operat*:ti,ab,kw OR postoperat*:ti,ab,kw OR postop:ti,ab,kw OR 'post operat*':ti,ab,kw OR 'post op':ti,ab,kw OR resect*:ti,ab,kw OR surgery:ti,ab,kw OR surgical*:ti,ab,kw OR surgeries:ti,ab,kw \| 4,661,605 \| \| #12 \| #7 OR #8 OR #9 OR #10 OR #11 \| 5,971,093 \| \| #13 \| #6 AND #12 \| 147,862 \| \| #14 \| 'esophagectomy'/exp \| 28,050 \| \| #15 \| 'gastrectomy'/exp \| 79177 \| \| #16 \| 'gastroenterostomy'/de \| 3,658 \| \| #17 \| esophagectom*:ti,ab,kw OR gastrectom*:ti,ab,kw OR gastroenterostom*:ti,ab,kw OR oesophagectom*:ti,ab,kw \| 79,232 \| \| #18 \| #14 OR #15 OR #16 OR #17 \| 116,308 \| \| #19 \| #13 OR #18 \| 212,733 \| \| #20 \| 'cardiorespiratory fitness'/de \| 9,094 \| \| #21 \| 'endurance training'/de OR 'resistance training'/de \| 34,960 \| \| #22 \| 'exercise'/de \| 358,897 \| \| #23 \| 'fitness'/de \| 44,746 \| \| #24 \| 'kinesiotherapy'/exp \| 104,275 \| \| #25 \| 'physiotherapy'/de \| 112,417 \| \| #26 \| exercis*:ti,ab,kw OR kinesiotherap*:ti,ab,kw OR 'physical function*':ti,ab,kw OR 'physical therap*':ti,ab,kw OR physiotherap*:ti,ab,kw OR 'physio therap*':ti,ab,kw OR 'training program*':ti,ab,kw \| 689,708 \| \| #27 \| (physical* NEAR/3 (activ* OR conditioning* OR fit OR inactiv* OR intervention* OR program*)):ab \| 231,333 \| \| #28 \| physical*:ti,kw AND (activ*:ti,kw OR conditioning*:ti,kw OR fit:ti,kw OR inactiv*:ti,kw OR intervention*:ti,kw OR program*:ti,kw) \| 101,813 \| \| #29 \| (fitness NEAR/3 (cardiorespirat* OR physical* OR training*)):ab \| 24,382 \| \| #30 \| fitness:ti,kw AND (cardiorespirat*:ti,kw OR physical*:ti,kw OR training*:ti,kw) \| 16,714 \| \| #31 \| (training NEAR/3 (endurance* OR intervent* OR physical*)):ab \| 32,723 \| \| #32 \| training:ti,kw AND (endurance*:ti,kw OR intervent*:ti,kw OR physical*:ti,kw) \| 22,580 \| \| #33 \| #20 OR #21 OR #22 OR #23 OR #24 OR #25 OR #26 OR #27 OR #28 OR #29 OR #30 OR #31 OR #32 \| 1,071,922 \| \| #34 \| #19 AND #33 \| 2,535 \| \| #35 \| #19 AND #33 AND [english]/lim \| 2,382 \| | |

3. Cochrane Library

| Interface: Wiley  Date of Search: 11 October 2023  Number of hits: 528 | Field labels   - ti,ab,kw = title, abstract and author keywords - NEAR/x = within x words, regardless of order - * = truncation of word for alternate endings |
| --- | --- |
| \| ID \| Search \| Hits \| \| --- \| --- \| --- \| \| #1 \| [mh "Esophageal Neoplasms"] \| 2349 \| \| #2 \| [mh ^"Gastrointestinal Neoplasms"] \| 729 \| \| #3 \| [mh ^"Stomach Neoplasms"] \| 3529 \| \| #4 \| ((abdomen OR abdominal OR esophag* OR gastric OR gastroesophage* OR gastrointestin* OR oesophag* OR stomach* ) AND (adenocarcinoma* OR carinoma* OR cancer* OR malignan* OR neoplas* OR tumor* OR tumour )):ti,kw \| 23097 \| \| #5 \| ((abdomen OR abdominal OR esophag* OR gastric OR gastroesophag* OR gastrointestin* OR oesophag* OR stomach* ) NEAR/3 (adenocarcinoma* OR carinoma* OR cancer* OR malignan* OR neoplas* OR tumor* OR tumour* )):ab \| 13194 \| \| #6 \| #1 OR #2 OR #3 OR #4 OR #5 \| 25994 \| \| #7 \| [mh ^"Digestive System Surgical Procedures"] \| 931 \| \| #8 \| [mh ^"Postoperative Care"] \| 5480 \| \| #9 \| [mh ^"Surgical Procedures, Operative"] \| 1278 \| \| #10 \| [mh /SU] \| 80116 \| \| #11 \| (operat* OR postoperat* OR postop OR post-operat* OR post-op OR resect* OR surgery OR surgical* OR surgeries ):ti,ab,kw \| 380660 \| \| #12 \| #7 OR #8 OR #9 OR #10 OR #11 \| 380660 \| \| #13 \| #6 AND #12 \| 12273 \| \| #14 \| [mh ^Esophagectomy] \| 881 \| \| #15 \| [mh ^Gastrectomy] \| 1596 \| \| #16 \| [mh ^Gastroenterostomy] \| 64 \| \| #17 \| (esophagectom* OR gastrectom* OR gastroenterostom* OR oesophagectom* ):ti,ab,kw \| 6282 \| \| #18 \| #14 OR #15 OR #16 OR #17 \| 6282 \| \| #19 \| #13 OR #18 \| 15055 \| \| #20 \| [mh ^Exercise] \| 25794 \| \| #21 \| [mh "Exercise Therapy"] \| 19761 \| \| #22 \| [mh "Physical Conditioning, Human"] \| 7338 \| \| #23 \| [mh "Physical Fitness"] \| 4467 \| \| #24 \| [mh ^"Physical Therapy Modalities"] \| 4777 \| \| #25 \| (exercis* OR kinesiotherap* OR ("physical" NEXT function*) OR ("physical" NEXT therap*) OR physiotherap* OR physio-therap* OR ("training" NEXT program*) ):ti,ab,kw \| 169485 \| \| #26 \| (physical* NEAR/3 (activ* OR conditioning* OR fit OR inactiv* OR intervention* OR program* )):ab \| 44892 \| \| #27 \| (physical* AND (activ* OR conditioning* OR fit OR inactiv* OR intervention* OR program* )):ti,kw \| 32588 \| \| #28 \| (fitness NEAR/3 (cardiorespirat* OR physical* OR training* )):ab \| 6263 \| \| #29 \| (fitness AND (cardiorespirat* OR physical* OR training* )):ti,kw \| 7160 \| \| #30 \| (training NEAR/3 (endurance* OR intervent* OR physical* )):ab \| 14501 \| \| #31 \| (training AND (endurance* OR intervent* OR physical* )):ti,kw \| 16672 \| \| #32 \| #20 OR #21 OR #22 OR #23 OR #24 OR #25 OR #26 OR #27 OR #28 OR #29 OR #30 OR #31 \| 201335 \| \| #33 \| #19 AND #32 \| 544 \| \| #34 \| #33 in Trials \| 543 \| \| #35 \| #34 in English \| 528 \| | |

4. Cinahl

| Interface: Ebsco  Date of Search: 11 October 2023  Number of hits: 255 | Field labels   - MH+ = exploded Cinahl Heading - MH = non exploded Cinahl Heading - TI = title - AB = abstract - Nx = within x words, regardless of order - * = truncation of word for alternate endings |
| --- | --- |
| \| # \| Query \| Results \| \| --- \| --- \| --- \| \| S1 \| (MH "Esophageal Neoplasms+") \| 9,400 \| \| S2 \| (MH "Gastrointestinal Neoplasms") \| 5,433 \| \| S3 \| (MH "Stomach Neoplasms") \| 12,811 \| \| S4 \| AB ((abdomen OR abdominal OR esophag* OR gastric OR gastroesophage* OR gastrointestin* OR oesophag* OR stomach*) N2 (adenocarcinoma* OR carinoma* OR cancer* OR malignan* OR neoplas* OR tumor* OR tumour)) \| 21,248 \| \| S5 \| TI ((abdomen OR abdominal OR esophag* OR gastric OR gastroesophage* OR gastrointestin* OR oesophag* OR stomach*) AND (adenocarcinoma* OR carinoma* OR cancer* OR malignan* OR neoplas* OR tumor* OR tumour)) \| 22,335 \| \| S6 \| S1 OR S2 OR S3 OR S4 OR S5 \| 38,815 \| \| S7 \| (MH "Surgery, Digestive System") \| 6,297 \| \| S8 \| (MH "Postoperative Care") \| 19,226 \| \| S9 \| (MH "Surgery, Operative") \| 26,564 \| \| S10 \| MW "SU" \| 433,587 \| \| S11 \| TI ( (operat* OR postoperat* OR postop OR post-operat* OR post-op OR resect* OR surgery OR surgical* OR surgeries ) ) OR AB ( (operat* OR postoperat* OR postop OR post-operat* OR post-op OR resect* OR surgery OR surgical* OR surgeries ) ) \| 667,969 \| \| S12 \| S7 OR S8 OR S9 OR S10 OR S11 \| 879,455 \| \| S13 \| S6 AND S12 \| 14,608 \| \| S14 \| (MH "Gastrectomy") \| 4,194 \| \| S15 \| (MH "Gastroenterostomy") \| 270 \| \| S16 \| TI ( (esophagectom* OR gastrectom* OR gastroenterostom* OR oesophagectom*) ) OR AB ( (esophagectom* OR gastrectom* OR gastroenterostom* OR oesophagectom*) ) \| 7,499 \| \| S17 \| S14 OR S15 OR S16 \| 8,631 \| \| S18 \| S13 OR S17 \| 19,141 \| \| S19 \| (MH "Exercise") \| 59,510 \| \| S20 \| (MH "Therapeutic Exercise+") \| 63,427 \| \| S21 \| (MH "Physical Fitness") OR (MH "Cardiorespiratory Fitness") \| 20,838 \| \| S22 \| (MH "Endurance Training") \| 842 \| \| S23 \| TI ( (exercis* OR kinesiotherap* OR "physical function*" OR "physical therap*" OR physiotherap* OR physio-therap* OR "training program*" ) ) OR AB ( (exercis* OR kinesiotherap* OR "physical function*" OR "physical therap*" OR physiotherap* OR physio-therap* OR "training program*" ) ) \| 222,001 \| \| S24 \| AB (physical* N3 (activ* OR conditioning* OR fit OR inactiv* OR intervention* OR program*)) \| 83,023 \| \| S25 \| TI (physical* AND (activ* OR conditioning* OR fit OR inactiv* OR intervention* OR program*)) \| 39,412 \| \| S26 \| AB (fitness N3 (cardiorespirat* OR physical* OR training*)) \| 8,908 \| \| S27 \| TI (fitness AND (cardiorespirat* OR physical* OR training*)) \| 4,995 \| \| S28 \| AB (training N3 (endurance* OR intervent* OR physical*)) \| 12,837 \| \| S29 \| TI (training AND (endurance* OR intervent* OR physical*)) \| 4,839 \| \| S30 \| S19 OR S20 OR S21 OR S22 OR S23 OR S24 OR S25 OR S26 OR S27 OR S28 OR S29 \| 348,787 \| \| S31 \| S18 AND S30 \| 274 \| \| S32 \| S31 **Narrow by Language:**- english \| 255 \| | |

5. PEDro

| Interface: pedro.org  Date of Search: 27 november 2023  Number of hits: 101 | Searched in   - Abstract & Title |
| --- | --- |
| "abdominal cancer"  "esophageal cancer"  "gastric cancer"  "oesophageal cancer"  "stomach cancer"  esophagectomy  gastrectomy  oesophagectomy | |

6. ClinicalTrials.gov

| Interface: clinicaltrials.gov  Date of Search: 11 October 2023  Number of hits: 783 | Searched in:   - Condition or disease - Other terms |
| --- | --- |
| Condition or disease:  (abdominal cancer* OR abdominal tumor* OR abdominal tumour* OR esophag* cancer* OR esophag* tumor* OR esophag* tumour* OR gastric cancer* OR gastric tumor* OR gastric tumour* OR gastroesophag* cancer* OR gastroesophag* tumor* OR gastroesophag* tumour* OR oesophag* cancer* OR oesophag* tumor* OR oesophag* tumour* OR stomach cancer* OR stomach tumor* OR stomach tumour* OR esophagectom* or gastrectom* or gastroenterostom* or oesophagectom*)AND  Other terms:  (exercis* OR fit OR fitness OR kinesiotherap* OR "physical activit*" OR "physical inactive*" OR "physical conditioning*" OR "physical function*" OR "physical intervention*" OR "physical program*" OR "physical therap*" OR physiothera* OR "physio-therap*")) | |

7. WHO ICTRP

| Interface: https://trialsearch.who.int/  Date of Search: 11 October 2023  Number of hits: 124 |  |
| --- | --- |
| (("abdominal cancer*" OR "abdominal tumor*" OR "abdominal tumour*" OR "esophag* cancer*" OR "esophag* tumor*" OR "esophag* tumour*" OR "gastric cancer*" OR "gastric tumor*" OR "gastric tumour*" OR "gastroesophag* cancer*" OR "gastroesophag* tumor*" OR "gastroesophag* tumour*" OR "oesophag* cancer*" OR "oesophag* tumor*" OR "oesophag* tumour*" OR "stomach cancer*" OR "stomach tumor*" OR "stomach tumour*" OR esophagectom* or gastrectom* or gastroenterostom* or oesophagectom*)  AND  (exercis* OR fit OR fitness OR kinesiotherap* OR "physical* activit*" OR "physica* inactive*" OR "physica* conditioning*" OR "physical* function*" OR "physical* intervention*" OR "physical* program*" OR "physical therap*" OR physiothera* OR "physio-therap*")) | |

**Excluded studies with reasons**

Protocols from trial registries that may have been eligible for inclusion, authors contacted, no response 3, 4, 5, 6, 7, 8, 9

Trial registration or additional publication of the included studies. 10, 11, 13, 14, 35, 40, 51

Wrong publication type: 1, 2, 15, 36, 37, 60, 61

Wrong population: 16, 23, 25, 45, 48, 52, 57, 62, 63

Wrong study design: 12, 19, 31, 34, 38, 41, 46, 49, 50, 53, 54, 56, 59

Wrong language: 39

Wrong study intervention duration: 42,

Wrong intervention 17, 18, 20, 21, 22, 24, 26, 27, 28, 29, 33, 43, 44, 47, 55, 58, 64, 65, 66

Wrong comparison: 30

From trial registries, withdrawn: 32

Choi et al. assessed quality of life using a 14-item, 5-point Likert scale, with negative items reversed. The total score ranges from 14 to 70, with higher scores indicating better QoL. To ensure consistency with other studies reporting QoL on a 0–100 scale, a linear transformation was applied. Initially, the reported mean item scores (3.78 in the exercise group and 3.16 in the control group) were converted to total scores on the 14–70 scale by multiplying by 14. This resulted in a total scores of 52.92 for the exercise group and 44.24 for the control group. Subsequently, these values were rescaled to the 0–100 scale using a standard formula: the minimum possible score (14) was subtracted from the observed score, divided by the range of the original scale (56), and multiplied by 100. This resulted in transformed mean scores of 69.5 for the exercise group and 54.0 for the control group, respectively. Standard deviations were adjusted using the same scale factor. The reported standard deviations (0.71 in the exercise group and 0.72 in the control group) were multiplied by 100 and divided by 56, resulting in transformed standard deviations of 1.27 for the exercise group and 1.29 for the control group, respectively. As this study used a different HRQL scale than the others in the meta-analysis, a standardised mean difference (SMD) model with a random-effects approach was employed to account for variability across instruments.


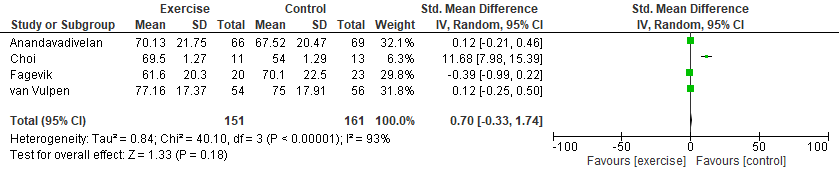


1. Fagevik Olsén M, Kjellby Wendt G, Hammerlid E, Smedh U. Effects of a Training Intervention after Thoraco-Abdominal Oesophagus Surgery—A Randomized Controlled Trial. Research Report Poster Presentation Number: RR-PO-01-05-Mon Monday 4 May 2015 12:15 Exhibit Halls 401–403. [WCPTCongress2015 Singapore, Conference Abstract
2. May AMA, Vulpen JV, Hiensch AE, Ruurda JP, Nieuwenhuijzen G, Kouwenhoven E, Groenendijk RPR, Peet DV, Hazebroek EJ, Rosman C, Wijnhoven BPL, Henegouwen MIVB, Laarhoven HWMV, Hillegersberg RV, Siersema P. Randomized clinical trial on the effect of a supervised exercise program on quality of life, fatigue, and fitness following esophageal cancer treatment (PERFECT study). Journal of Clinical Oncology. 2020;38(15):suppl.12055. DOI: 10.1200/JCO.2020.38.15-suppl.12055.
3. Taipei Veterans General Hospital, Taiwan. The Effect of Home Based Walking Exercise on Fatigue, Anxiety, Depression, Sleep Quality, Circadian Rhythms and Quality of Life in Patients With Gastric Cancer Undergoing Gastrectomy. ClinicalTrials.gov Identifier: NCT04593134. Taipei Veterans General Hospital, Taiwan. Last Update Posted 2021-12-21. Contact: Pei-Shan Ho, BSN
4. A Randomized Controlled Study of the Out-patient Rehabilitation Intervention following Thoracic Esophageal Cancer Surgery - A Randomized Controlled Study of the Out-patient Rehabilitation Intervention following Thoracic Esophageal Cancer Surgery. [JPRN-UMIN000049498]. Register: JPRN. Prospective Registration: Yes. Date of Registration: 15/11/2022. Last Refreshed on: 17 October 2023. Study Type: Interventional. Study Design: Parallel Randomized. Phase: Phase III. Lead Principal Investigator: Hiroshi Sato.
5. The Feasibility of Liuzijue Qigong (LQG) for the Rehabilitation of Postoperative Patients with Esophageal Resection - a Randomized Controlled Trial. [ChiCTR2100050382]. Register: ChiCTR. Prospective Registration: Yes. Date of Registration: 2021-08-27. Last Refreshed on: 25 April 2022. Study Type: Interventional Study. Study Design: Parallel. Recruitment Status: Recruiting. Primary Sponsor: Li Junmin, Shanghai Xuhui Central Hospital, 966 Huaihai Middle Road, Xuhui District, Shanghai.
6. Effect of postoperative immediate breathing exercise in postanesthesia care unit after extubation on pulmonary complications after esophageal cancer surgery. [ChiCTR2100052851]. Register: ChiCTR. Prospective Registration: No. Date of Registration: 2021-11-06. Last Refreshed on: 23 August 2022. Study Type: Interventional Study. Study Design: Parallel. Phase: 0. Recruitment Status: Recruiting. Primary Sponsor: Cancer Center of Sun Yat-Sen University, 651 Dongfeng Road East, Yuexiu District, Guangzhou, Guangdong.
7. Clinical study of progressive elastic resistance training on lung function and exercise tolerance in post-operated patients with thoracic tumor. [ChiCTR1800020028]. Register: ChiCTR. Prospective Registration: Yes. Date of Registration: 2018-12-12. Last Refreshed on: 18 December 2018. Study Type: Interventional study. Study Design: Randomized parallel controlled trial. Phase: Other. Recruitment Status: Recruiting. Primary Sponsor: First Affiliated Hospital of Zhejiang University School of Medicine, 79 Qingchun Road, Hangzhou, Zhejiang, China.
8. Clinical study of progressive elastic resistance training on lung function and exercise tolerance in post-operated patients with thoracic tumor. [ChiCTR1800020028]. Register: ChiCTR. Prospective Registration: Yes. Date of Registration: 2018-12-12. Last Refreshed on: 18 December 2018. Study Type: Interventional study. Study Design: Randomized parallel controlled trial. Phase: Other. Recruitment Status: Recruiting. Countries of Recruitment: China. Primary Sponsor: First Affiliated Hospital of Zhejiang University School of Medicine, 79 Qingchun Road, Hangzhou, Zhejiang, China.
9. Post-operative high intensity interval training in patients undergoing major foregut cancer surgery: A randomised controlled trial. [ACTRN12620000315910]. Register: ANZCTR. Prospective Registration: No. Date of Registration: 06/03/2020. Last Refreshed on: 27 March 2023. Study Type: Interventional. Study Design: Purpose: Prevention; Allocation: Randomised controlled trial; Masking: Open (masking not used); Assignment: Parallel; Type of Endpoint: Efficacy. Phase: Not Applicable. Recruitment Status: Recruiting. Countries of Recruitment: Australia. Primary Sponsor: St Vincent's Hospital, Melbourne, 41 Victoria Parade, Fitzroy VIC 3065 Australia.
10. Physical ExeRcise Following Esophageal Cancer Treatment (PERFECT) Study: a randomized clinical trial. [NTR5045]. Register: Netherlands Trial Register. Prospective Registration: Yes. Date of Registration: 2015-01-19. Last Refreshed on: 3 April 2023. Study Type: Interventional. Study Design: Randomized controlled trial, Single blinded (masking used), Active, Parallel. Recruitment Status: Completed. Primary Sponsor: University Medical Center Utrecht, Postal address STR 6.131, P.O. Box 85500, 3508 GA Utrecht, The Netherlands.
11. Physical Activity Intervention Trial to Mitigate Muscle Loss and Increase Muscle Strength in Esophageal Cancer Survivors. [PA study]. Register: ClinicalTrials.gov, NCT02786524. [Study Start: 2016-01, Primary Completion: 2020-06, Study Completion: 2020-06]. Conditions: Oesophageal Cancer. Intervention: Behavioral - Physical activity intervention group. Interventional. Sponsor: Karolinska Institutet. Principal Investigator: Pernilla Lagergren, Professor, Karolinska Institutet. Last Update Posted: 2020-12-14. [Online].
12. Simonsen C, Thorsen-Streit S, Sundberg A, Djurhuus SS, Mortensen CE, Qvortrup C, Pedersen BK, Svendsen LB, de Heer P, Christensen JF. Effects of high-intensity exercise training on physical fitness, quality of life and treatment outcomes after oesophagectomy for cancer of the gastro-oesophageal junction: PRESET pilot study. BJS Open. 2020 Oct;4(5):855-864. doi: 10.1002/bjs5.50337. Epub 2020 Aug 28. PMID: 32856785; PMCID: PMC7528530.
13. van Vulpen JK, Siersema PD, van Hillegersberg R, Nieuwenhuijzen GAP, Kouwenhoven EA, Groenendijk RPR, van der Peet DL, Hazebroek EJ, Rosman C, Schippers CCG, Steenhagen E, Peeters PHM, May AM. Physical ExeRcise Following Esophageal Cancer Treatment (PERFECT) study: design of a randomized controlled trial. BMC Cancer. 2017 Aug 18;17(1):552. doi: 10.1186/s12885-017-3542-8. PMID: 28821284; PMCID: PMC5562993.
14. Hiensch A, Steenhagen E, van Vulpen JK, Ruurda JP, Nieuwenhuijzen GAP, Kouwenhoven EA, Groenendijk RPR, van der Peet DL, Rosman C, Wijnhoven BPL, van Berge Henegouwen MI, van Laarhoven HWM, van Hillegersberg R, Siersema PD, May AM. Effects of exercise after oesophagectomy on body composition and adequacy of energy and protein intake: PERFECT multicentre randomized controlled trial. BJS Open. 2023 Jul 10;7(4):zrad057. doi: 10.1093/bjsopen/zrad057. PMID: 37527034; PMCID: PMC10392959.
15. Simonsen C, Thorsen-Streit S, Sundberg A, Sigmundsdóttir Djurhuus S, Ehlers Mortensen C, Qvortrup C, Klarlund Pedersen B, Svendsen L, de Heer P, Frank Christensen J. Effects of exercise training after esophagectomy on physical fitness, quality of life and treatment outcomes. Diseases of the Esophagus. 2020;33(Suppl 1):30. DOI: 10.1093/dote/doaa087.78.
16. Sichuan Cancer Hospital. Construction and application of perioperative progressive exercise rehabilitation management model in patients with abdominal malignant tumor. [ChiCTR2300074335]. Register: ChiCTR. Prospective Registration: Yes. Date of Registration: 2023-08-03. Last Refreshed on: 7 August 2023. Study Type: Interventional study. Study Design: Parallel. Phase: 0. Recruitment Status: Pending. Primary Sponsor: Sichuan Cancer Hospital, Address: 55 Section 4, Renmin South Road, Wuhou District, Chengdu City, Sichuan Province 610041, China.
17. Rehabilitation and Recovery for Persons With Esophageal or Gastric Cancer. ClinicalTrials.gov Identifier: NCT05698992. Sponsor: Region Skane. Study Type: Observational [Patient Registry]. Study Start (Actual): 2023-02-27. Primary Completion (Estimated): 2026-12-31. Study Completion (Estimated): 2027-12-31. Enrollment (Estimated): 100. Last Update Posted: 2023-03-13. Contacts: Wenche Melander, PhD-student Collaborators: Karolinska Institutet. Study Registration Dates: First Submitted: 2022-12-02; First Submitted that Met QC Criteria: 2023-01-16; First Posted: 2023-01-26. Study Record Updates: Last Update Submitted that met QC Criteria: 2023-03-09; Last Update Posted: 2023-03-13; Last Verified: 2023-03.
18. Teleprehabilitation for Surgical Cancer Patients. ClinicalTrials.gov Identifier: NCT04799561. Sponsor: McGill University Health Centre/Research Institute of the McGill University Health Centre. Study Contact: Francesco Carli, MD, Last Update Posted: 2021-09-30.
19. ReStOre@Home: Feasibility Study of a 12-week Multidisciplinary Telehealth Rehabilitation Programme for Survivors of Upper Gastrointestinal (UGI) Cancer (ReStOre@Home). ClinicalTrials.gov Identifier: NCT04603339. Sponsor: University of Dublin, Trinity College. Information provided by Prof Juliette Hussey, University of Dublin, Trinity College (Responsible Party). Last Update Posted: 2022-04-13. Study Start (Actual): 2021-05-01. Primary Completion (Actual): 2021-10-30. Study Completion (Actual): 2022-01-01. Enrollment (Actual): 12. Study Type: Interventional. Phase: Not Applicable.
20. Effect of Smart Phone App-based Human Coaching Program in Gastrectomized Patients. ClinicalTrials.gov Identifier: NCT04394585. Sponsor: National Cancer Center, Korea. Information provided by Bang Wool Eom, National Cancer Center, Korea (Responsible Party). Last Update Posted: 2023-03-27. Study Start (Actual): 2020-04-20. Primary Completion (Actual): 2022-11-02. Study Completion (Estimated): 2023-12. Enrollment (Estimated): 180. Study Type: Interventional. Phase: Not Applicable. Study Contact: Bang Wool Eom, PhD.
21. Royal College of Surgeons, Ireland. Pre- and Post-Operative Exercise Program in Upper Gastrointestinal Cancers (PERIOP-OG). ClinicalTrials.gov Identifier: NCT03807518. Sponsor: Royal College of Surgeons, Ireland. Information provided by Royal College of Surgeons, Ireland (Responsible Party). Last Update Posted: 2021-03-09. Primary Completion (Actual): 2020-12-19. Study Completion (Actual): 2021-03-01. Enrollment (Actual): 72. Study Type: Interventional. Phase: Not Applicable.
22. Enhanced Recovery After Surgery Program for Gastric Cancer: a Multi-center Study (ERASG1). ClinicalTrials.gov Identifier: NCT03121729. Sponsor: Jinling Hospital, China. Information provided by Jian ZHAO, Jinling Hospital, China (Responsible Party). Last Update Posted: 2018-04-30. Study Start (Actual): 2017-06-21. Primary Completion (Estimated): 2018-08-31. Study Completion (Estimated): 2018-12. Enrollment (Estimated): 1000. Study Type: Interventional. Phase: Not Applicable. Study Contact: Name - Zhi-Wei JIANG, Ph.D.
23. The Assessment of the Feasibility of a Home Based Exercise Programme in the Older Patient Following Major Surgery (POETold). ClinicalTrials.gov Identifier: NCT03064308. Sponsor: University of Nottingham. Information provided by University of Nottingham (Responsible Party). Last Update Posted: 2019-04-17. Study Start (Actual): 2017-06-26. Primary Completion (Actual): 2018-07-26. Study Completion (Actual): 2018-07-27. Enrollment (Actual): 11. Study Type: Interventional. Phase: Not Applicable. Collaborators: No information provided.
24. Hershey Medical Center. Resilience and Exercise in Advanced Cancer Treatment (REACT). ClinicalTrials.gov Identifier: NCT02680990. Sponsor: Milton S. Hershey Medical Center. Information provided by Amanda Cooper, MD, Milton S. Hershey Medical Center (Responsible Party). Last Update Posted: 2018-02-28. Study Start: 2015-12. Primary Completion (Actual): 2017-07. Study Completion (Actual): 2017-07. Enrollment (Actual): 14. Study Type: Interventional. Phase: Not Applicable.
25. Effect of a Self-designed MET Exercise Intervention on Cancer-related Fatigue in Patients With Gastric Cancer (SMEE). ClinicalTrials.gov Identifier: NCT05401045. Sponsor: Ruijin Hospital. Information provided by Lei Huang, Ruijin Hospital (Responsible Party). Last Update Posted: 2023-01-27. Study Start: 2023-01-20. Primary Completion (Estimated): 2023-06. Study Completion (Estimated): 2023-07. Enrollment (Estimated): 119. Study Type: Interventional. Phase: Not Applicable. Study Contact: Name: Jun Zhang, PhD.
26. Rehabilitation Strategies Following Oesophagogastric and Hepatopancreaticobiliary Cancer (RESTORE II). ClinicalTrials.gov Identifier: NCT03958019. Sponsor: University of Dublin, Trinity College. Information provided by Prof Juliette Hussey, University of Dublin, Trinity College (Responsible Party). Last Update Posted: 2022-04-13. Study Start: 2022-01-25. Primary Completion (Estimated): 2024-06-01. Study Completion (Estimated): 2024-12-01. Enrollment (Estimated): 120. Study Type: Interventional. Phase: Not Applicable. Study Contact: Name: Linda O'Neill, PhD.
27. Pre- and Post-Operative Exercise Program in Upper Gastrointestinal Cancers (PERIOP-OG). ClinicalTrials.gov Identifier: NCT03807518. Sponsor: Royal College of Surgeons, Ireland. Information provided by Royal College of Surgeons, Ireland (Responsible Party). Last Update Posted: 2021-03-09. Study Start (Actual): 2019-03-01. Primary Completion (Actual): 2020-12-19. Study Completion (Actual): 2021-03-01. Enrollment (Actual): 72. Study Type: Interventional. Phase: Not Applicable.
28. Effects of Increased Physical Activity Before Thoracoabdominal Esophageal Surgery. ClinicalTrials.gov Identifier: NCT03452319. Sponsor: Göteborg University. Information provided by Göteborg University (Responsible Party). Last Update Posted: 2023-10-02. Study Start (Actual): 2017-12-01. Primary Completion (Actual): 2022-12-31. Study Completion (Actual): 2023-08-30. Enrollment (Actual): 100. Study Type: Interventional. Phase: Not Applicable.
29. Rehabilitative Strategies Following Oesophageal Cancer (ReStOre). ClinicalTrials.gov Identifier: NCT03314311. Sponsor: University of Dublin, Trinity College. Information provided by Prof Juliette Hussey, University of Dublin, Trinity College (Responsible Party). Last Update Posted: 2017-10-19. Study Start: 2014-10. Primary Completion (Actual): 2017-06. Study Completion (Actual): 2017-06. Enrollment (Actual): 44. Study Type: Interventional. Phase: Not Applicable.
30. Effects of a Training Intervention After Thoracoabdominal Oesophagus Surgery. ClinicalTrials.gov Identifier: NCT02335970. Sponsor: Göteborg University. Information provided by Göteborg University (Responsible Party). Last Update Posted: 2020-01-06. Study Start (Actual): 2005-09-05. Primary Completion (Actual): 2011-06. Study Completion (Actual): 2011-06. Enrollment (Actual): 64. Study Type: Interventional. Phase: Not Applicable.
31. An Effect of an Individualized Physical Activity Intervention for Gastric Cancer Patient Undergoing Minimally Invasive Gastrectomy: a Phase III, Prospective Randomized Controlled Trial (Gastric cancer). ClinicalTrials.gov Identifier: NCT02120885. Sponsor: Yonsei University. Information provided by Yonsei University (Responsible Party). Last Update Posted: 2019-01-22. Study Start (Actual): 2014-03-14. Primary Completion (Actual): 2017-07-10. Study Completion (Actual): 2017-07-10. Enrollment (Actual): 70. Study Type: Interventional. Phase: Phase 3.
32. Mobility and Activity Training (MAT) to Optimize Outcomes for Older Adult Abdominal Surgery Patients (MAT). ClinicalTrials.gov Identifier: NCT02007876. Sponsor: University of Michigan. Information provided by Michael Englesbe, University of Michigan (Responsible Party). Last Update Posted: 2016-02-03. Study Start: 2013-06. Primary Completion (Actual): 2016-01. Study Completion (Actual): 2016-01. Enrollment (Actual): 0. Study Type: Interventional. Phase: Not Applicable.
33. Research on remote exercise support for esophageal cancer patients. Effects of a remote physical activity intervention for esophageal cancer patients undergoing neoadjuvant chemotherapy: a randomized controlled trial. JPRN-jRCT1062220049. Registered on 20/08/2022. Primary sponsor: Noma Tomohiro. Last refreshed on 17 October 2023. Date of first enrollment: 01/08/2022. Target sample size: 58. Recruitment status: Recruiting. Study type: Interventional. Study design: Randomized controlled trial, open (masking not used), no treatment control/standard of care control, parallel assignment, treatment purpose. Phase 2.
34. Sellar CM, Courneya KS. Physical activity and gastrointestinal cancer survivorship. Recent Results Cancer Res. 2011;186:237-53. doi: 10.1007/978-3-642-04231-7_10. PMID: 21113767.
35. van Vulpen JK, Witlox L, Methorst-de Haan AC, Hiensch AE, van Hillegersberg R, Ruurda JP, Nieuwenhuijzen GAP, Kouwenhoven EA, Siersema PD, May AM. Perceived facilitators and barriers by esophageal cancer survivors participating in a post-treatment exercise program. Support Care Cancer. 2023 May 6;31(6):320. doi: 10.1007/s00520-023-07769-5. PMID: 37148366; PMCID: PMC10164010.
36. Loughney, L., Tully, R., Bolger, J. C., Sorensen, J., McAnena, O., Collins, C. G., Carroll, P. A., & Arumuga, M. (2023). The effect of a pre- and post-operative exercise programme versus standard care on physical fitness of patients with oesophageal and gastric cancer undergoing neoadjuvant treatment prior to surgery (The PERIOP-OG Trial): A Randomised controlled trial [Conference Abstract]. In Proceedings of the European Colorectal Congress, St.Gallen, Switzerland, December 3-6, 2023.
37. Li YC. The effect of a community-based rehabilitation program on post-operative cancer-related fatigue and quality of life in esophageal elderly patients undergoing chemotherapy. In: Abstracts from the Third Chinese Congress on Gerontology and Health Industry. The First Affiliated Clinical College of Changzhi Medical College, Changzhi, China.
38. O’Neill L, Guinan E, Doyle SL, Elliott JA, O’Sullivan J, Reynolds JV, Hussey J. Rehabilitation strategies following esophageal cancer (the ReStOre trial): a feasibility study. Dis Esophagus. 2017 May 9;30(5):1–8. doi: 10.1093/dote/dow012. PMCID: PMC6036658.
39. Choi JY, Kang HS. [Effects of a home-based exercise program for patients with stomach cancer receiving oral chemotherapy after surgery]. J Korean Acad Nurs. 2012 Feb;42(1):95-104. Korean. doi: 10.4040/jkan.2012.42.1.95. PMID: 22410606.
40. van Vulpen, J.K., Siersema, P.D., van Hillegersberg, R. *et al.* Physical ExeRcise Following Esophageal Cancer Treatment (PERFECT) study: design of a randomized controlled trial. *BMC Cancer* **17**, 552 (2017). <https://doi.org/10.1186/s12885-017-3542-8>
41. Tanaka K, Taoda A, Kashiwagi H. Resistance Exercise After Laparoscopic Surgery Enhances Improvement in Exercise Tolerance in Geriatric Patients With Gastrointestinal Cancer. Cureus. 2021 Jun 5;13(6):e15454. doi: 10.7759/cureus.15454. PMID: 34258117; PMCID: PMC8256392.
42. Steffens D, Solomon MJ, Beckenkamp PR, Koh CE, Yeo D, Sandroussi C; Fit-4-Home Collaborators; Hancock MJ. Individualised, targeted step count intervention following gastrointestinal cancer surgery: The Fit-4-Home randomised clinical trial. ANZ J Surg. 2022 Apr;92(4):703-711. doi: 10.1111/ans.17212. Epub 2021 Sep 22. PMID: 34553480.
43. O'Neill LM, Guinan E, Doyle SL, Bennett AE, Murphy C, Elliott JA, OʼSullivan J, Reynolds JV, Hussey J. The RESTORE Randomized Controlled Trial: Impact of a Multidisciplinary Rehabilitative Program on Cardiorespiratory Fitness in Esophagogastric cancer Survivorship. Ann Surg. 2018 Nov;268(5):747-755. doi: 10.1097/SLA.0000000000002895. PMID: 30004915.
44. Prehabilitation and Rehabilitation for Patients With Oesophageal Cancer (PRESO) [ClinicalTrials.gov Identifier NCT04894149]. Sponsor: Gabriella Alexandersson von Döbeln. Information provided by Gabriella Alexandersson von Döbeln, Karolinska University Hospital (Responsible Party). Last Update Posted: 2021-05-20. Study Start (Actual): 2021-04-22. Primary Completion (Estimated): 2023-01. Study Completion (Estimated): 2023-05. Enrollment (Estimated): 25. Study Type: Interventional. Phase: Not Applicable. Study Contact: Name - Gabriella A von Döbeln, MD, PhD. Sponsor - Gabriella Alexandersson von Döbeln.
45. Effectiveness of a Rehabilitation Program in Improving Quality of Life in Patients With Esophageal Cancer [ClinicalTrials.gov Identifier NCT03161535]. Sponsor: Taipei Veterans General Hospital, Taiwan. Information provided by Taipei Veterans General Hospital, Taiwan (Responsible Party). Last Update Posted: 2023-08-23. Study Start (Actual): 2017-09-19. Primary Completion (Estimated): 2024-07. Study Completion (Estimated): 2024-07. Enrollment (Actual): 85. Study Type: Interventional. Phase: Not Applicable. Sponsor: Taipei Veterans General Hospital, Taiwan.
46. PeRioperative Study of Exercise Training in Patients With Operable Cancer of the Gastroesophageal Junction (PRESET) [ClinicalTrials.gov Identifier NCT02722785]. Sponsor: Jesper Frank Christensen, PhD. Information provided by Jesper Frank Christensen, PhD, Rigshospitalet, Denmark (Responsible Party). Last Update Posted: 2018-08-15. Study Start (Actual): 2016-04. Primary Completion (Actual): 2017-10. Study Completion (Actual): 2018-05. Enrollment (Estimated): 40. Study Type: Interventional. Phase: Not Applicable. Sponsor: Jesper Frank Christensen, PhD.
47. An Effect of an Individualized Physical Activity Intervention for Gastric Cancer Patient Undergoing Minimally Invasive Gastrectomy: a Phase III, Prospective Randomized Controlled Trial (Gastric cancer) [ClinicalTrials.gov Identifier NCT02120885]. Sponsor: Yonsei University. Information provided by Yonsei University (Responsible Party). Last Update Posted: 2019-01-22. Study Start (Actual): 2014-03-14. Primary Completion (Actual): 2017-07-10. Study Completion (Actual): 2017-07-10. Enrollment (Actual): 70. Study Type: Interventional. Phase: Phase 3. Sponsor: Yonsei University.
48. Exercise in Improving Mobility and Reducing Fatigue and/or Weakness in Older Cancer Survivors [ClinicalTrials.gov Identifier NCT00335491]. Sponsor: University of Utah. Information provided by Paul LaStayo, University of Utah (Responsible Party). Last Update Posted: 2011-09-30. Study Start: 2006-03. Primary Completion (Actual): 2010-03. Study Completion (Actual): 2010-03. Enrollment (Actual): 49. Study Type: Interventional. Phase: Not Applicable. Sponsor: University of Utah.
49. Assessment of quality of life and adherence to chemotherapy according to muscle mass and amount of exercise in patients receiving adjuvant chemotherapy after gastrectomy. 2022 Jun 6. Institutional Review Board Number: NCC2022-0160. [Internet]. Goyang-si, Gyeonggi-do: National Cancer Center; [cited 2023 Nov 27].
50. Perioperative nutrition support and exercise for preventing skeletal muscle loss in patients with esophageal cancer undergoing esophagectomy: a prospective study. Unique ID: UMIN000042743. 2020 Dec 14 [Last Modified 2023 Jun 14 17:25:47]. Lead Principal Investigator: Kazutaka Obama. Kyoto University, Department of Surgery.
51. Hiensch A, Steenhagen E, van Vulpen JK, Ruurda JP, Nieuwenhuijzen GAP, Kouwenhoven EA, Groenendijk RPR, van der Peet DL, Rosman C, Wijnhoven BPL, van Berge Henegouwen MI, van Laarhoven HWM, van Hillegersberg R, Siersema PD, May AM. Effects of exercise after oesophagectomy on body composition and adequacy of energy and protein intake: PERFECT multicentre randomized controlled trial. BJS Open. 2023 Jul 10;7(4):zrad057. doi: 10.1093/bjsopen/zrad057. PMID: 37527034; PMCID: PMC10392959.
52. Herrstedt A, Bay ML, Simonsen C, Sundberg A, Egeland C, Thorsen-Streit S, Djurhuus SS, Magne Ueland P, Midttun Ø, Pedersen BK, Bo Svendsen L, de Heer P, Christensen JF, Hojman P. Exercise-mediated improvement of depression in patients with gastro-esophageal junction cancer is linked to kynurenine metabolism. Acta Oncol. 2019 May;58(5):579-587. doi: 10.1080/0284186X.2018.1558371. Epub 2019 Jan 30. PMID: 30696326.
53. Guinan, E.M., Doyle, S.L., O’Neill, L. *et al.* Effects of a multimodal rehabilitation programme on inflammation and oxidative stress in oesophageal cancer survivors: the ReStOre feasibility study. *Support Care Cancer* **25**, 749–756 (2017). <https://doi.org/10.1007/s00520-016-3455-0>.
54. Cho I, Son Y, Song S, Bae YJ, Kim YN, Kim HI, Lee DT, Hyung WJ. Feasibility and Effects of a Postoperative Recovery Exercise Program Developed Specifically for Gastric Cancer Patients (PREP-GC) Undergoing Minimally Invasive Gastrectomy. J Gastric Cancer. 2018 Jun;18(2):118-133. doi: 10.5230/jgc.2018.18.e12. Epub 2018 Apr 25. PMID: 29984062; PMCID: PMC6026706.
55. Chang YL, Tsai YF, Hsu CL, Chao YK, Hsu CC, Lin KC. The effectiveness of a nurse-led exercise and health education informatics program on exercise capacity and quality of life among cancer survivors after esophagectomy: A randomized controlled trial. Int J Nurs Stud. 2020 Jan;101:103418. doi: 10.1016/j.ijnurstu.2019.103418. Epub 2019 Sep 11. PMID: 31670173.
56. Bennett AE, O'Neill L, Connolly D, Guinan EM, Boland L, Doyle SL, O'Sullivan J, Reynolds JV, Hussey J. Patient experiences of a physiotherapy-led multidisciplinary rehabilitative intervention after successful treatment for oesophago-gastric cancer. Support Care Cancer. 2018 Aug;26(8):2615-2623. doi: 10.1007/s00520-018-4112-6. Epub 2018 Feb 18. PMID: 29455302.
57. Arslan E, Gezer N. The effect of discharge training developed based on nursing interventions classification (NIC) on surgical recovery in oncology patients: Randomized controlled trial - A pilot study. Nurs Open. 2023 Feb;10(2):1151-1162. doi: 10.1002/nop2.1555. Epub 2022 Dec 25. PMID: 36567264; PMCID: PMC9834180.
58. SupPoRtive Exercise Programmes for Accelerating REcovery after major ABdominal Cancer surgery trial (PREPARE-ABC): Pilot phase of a multicentre randomised controlled trial. *Colorectal Dis*. 2021; 23: 3008–3022
59. Aceto P, Congedo E, Cardone A, Zappia L, De Cosmo G. Postoperative management of elective esophagectomy for cancer. Rays. 2005 Oct-Dec;30(4):289-94. PMID: 16792002.
60. Podium Videos. In: Surgical Endoscopy (SURG ENDOSC), Denver, Colorado, 16-19 March 2022. 2022 May; 36(Suppl 1): 248-264. DOI: 10.1007/s00464-022-09220-y. MEDLINE PMID: NLM35441866.
61. Coebergh JW, Janssen-Heijnen ML, Post PN, Razenberg PP. Serious co-morbidity among unselected cancer patients newly diagnosed in the southeastern part of The Netherlands in 1993-1996. J Clin Epidemiol. 1999;52(12):1131-6.
62. Chen H, Huang M, Zhou J, Zhang X, Chen S, Liu C, Zhang K, Li Y, Zhang Y, Huang C. Enhancement of Pulmonary Function and Reduction of Complications Through EIT-Guided Yoga Breathing Exercise After Esophagectomy. Med Sci Monit. 2024 Jul 1;30:e942954. doi: 10.12659/MSM.942954. PMID: 38949992; PMCID: PMC11305053.
63. Xin X, Huang L, Pan Q, Zhang J, Hu W. The effect of self-designed metabolic equivalent exercises on cancer-related fatigue in patients with gastric cancer: A randomized controlled trial. Cancer Med. 2024 May;13(9):e7085. doi: 10.1002/cam4.7085. PMID: 38716637; PMCID: PMC11077428.
64. Lee JY, Oh EG, Jang Y, Lee J, Hyung W, Kim YC. Effectiveness of self-management program for gastric cancer patients: A randomized controlled trial comparing gain vs. loss message framing. Patient Educ Couns. 2024 Nov;128:108364. doi: 10.1016/j.pec.2024.108364. Epub 2024 Jul 15. PMID: 39047331.
65. Loughney L, Bolger J, Tully R, Sorensen J, Bambrick M, Carroll PA, Arumugasamy M, Murphy TJ, McCaffrey N, Robb WB; PERIOP-OG Working Group. The effect of a pre-operative and post-operative exercise programme versus standard care on physical fitness of patients with oesophageal and gastric cancer undergoing neoadjuvant treatment prior to surgery (the PERIOP-OG trial): a randomized controlled trial. Int J Surg. 2024 Oct 1;110(10):6632-6646. doi: 10.1097/JS9.0000000000001663. PMID: 38935085; PMCID: PMC11487022.
66. Bausys, A., Luksta, M., Anglickiene, G., Maneikiene, V.V., Kryzauskas, M., Rybakovas, A., Dulskas, A., Kuliavas, J., Stratilatovas, E., Macijauskiene, L., Simbelyte, T., Celutkiene, J., Jamontaite, I.E., Cirtautas, A., Lenickiene, S., Petrauskiene, D., Cikanaviciute, E., Gaveliene, E., Klimaviciute, G., Rauduvyte, K., Bausys, R., Strupas, K., 2023. Effect of home-based prehabilitation on postoperative complications after surgery for gastric cancer: randomized clinical trial. British Journal of Surgery 110, 1800–1807.. https://doi.org/10.1093/bjs/znad312
